# Botanical Extracts and Antagonistic Fungi for the Sustainable Management of Wheat Blast Caused by *Pyricularia oryzae Triticum* Pathotype

**DOI:** 10.3390/plants15213331

**Published:** 2026-10-28

**Authors:** Sergio Iván Martinez, Verónica Fabiana Consolo, Cecilia Smirnoff, Analía Edith Perelló

**Affiliations:** 1 Consejo Nacional de Investigaciones Científicas y Técnicas (CONICET), Buenos Aires C1425, Argentina; ivan.martinez.19912@gmail.com (S.I.M.); consolo.vf@gmail.com (V.F.C.); 2 Facultad de Ciencias Agrarias y Forestales (FCAyF), Universidad Nacional de La Plata (UNLP), La Plata, Buenos Aires CP1900, Argentina; 3 Instituto de Investigaciones en Biodiversidad y Biotecnología (INBIOTEC-CONICET), Fundación para Investigaciones Biológicas Aplicadas (FIBA), Mar del Plata B7600, Argentina; 4 Facultad de Ingeniería y Ciencias Agrarias (FICA), Pontificia Universidad Católica Argentina (UCA), Buenos Aires C1107, Argentina; ceciliasmirnoff@uca.edu.ar; 5 Facultad de Agronomía, Universidad de Buenos Aires (UBA), Buenos Aires C1417, Argentina

**Keywords:** wheat blast, *Pyricularia oryzae Triticum* pathotype, *Allium sativum*, *Trichoderma harzianum*, *Epicoccum nigrum*, disease suppression

## Abstract

Wheat blast, caused by *Pyricularia oryzae Triticum* pathotype (PoT), is a major threat to wheat production and highlights the need for sustainable alternatives to conventional fungicides. This study evaluated the antifungal, growth-related, and disease-suppressive effects of botanical extracts from *Lippia alba* and *Allium sativum* and the antagonistic fungi *Trichoderma harzianum* and *Epicoccum nigrum* against PoT under in vitro and in vivo conditions. In dual-culture and poisoned-food assays, *A. sativum* extract (5 mL) and *T. harzianum* (TH-FCCT-1155-4) strongly inhibited PoT, completely suppressing conidiation and reducing mycelial growth by more than 90% and 58%, respectively. Seed treatments with *A. sativum*, *T. harzianum*, and *E. nigrum* were non-phytotoxic and were associated with higher values for several seedling growth parameters in PoT-infected seedlings, including leaf number, stem thickness, and dry biomass, while reducing seed-to-seedling disease transmission. Foliar applications at the seedling (GS 1.5) and anthesis (GS 6.0) stages reduced disease development in several cultivar–isolate combinations, although treatment responses varied among PoT isolates and wheat cultivars. At anthesis, treatment effects on spike disease severity and thousand-grain weight (TGW) also varied among cultivar–isolate combinations. In contrast, *L. alba* showed phytotoxic effects at 1000 ppm and did not consistently reduce spike disease severity when applied at anthesis. RT-qPCR analysis showed that garlic seed treatment was associated with higher transcript abundance of the defense-related genes PR5 and APX at specific sampling times between 48 and 72 h post-inoculation. Overall, these results identify *A. sativum*, *T. harzianum*, and *E. nigrum* as candidates for further evaluation in standardized greenhouse and field studies for wheat blast management and provide evidence that their effects may involve direct antifungal activity and, in the case of *A. sativum*, modulation of host defense responses.

## 1. Introduction

Wheat blast, caused by *Pyricularia oryzae Triticum* pathotype (PoT), is a destructive disease that threatens wheat production and remains difficult to manage. Current control strategies rely largely on synthetic fungicides, although their effectiveness can vary and their intensive use raises environmental and health concerns. Integrated management combining resistant cultivars with chemical and complementary control measures is therefore required. However, effective sources of resistance remain limited, and although the 2NS/2AS translocation has been associated with increased resistance, its expression can vary depending on wheat genetic background and pathogen isolate [1,2,3,4]. Fungicides such as triazole-based products and mixtures have shown efficacy against PoT, but the need for sustainable and complementary disease-management strategies remains evident [5,6].

Biorational approaches based on plant-derived compounds and beneficial microorganisms represent promising alternatives or complements to conventional fungicides. Their potential extends beyond direct pathogen inhibition and may involve antibiosis, competition, mycoparasitism, and activation of plant defense responses, while some microbial agents can also promote plant growth and improve plant performance [7,8]. Botanical extracts are particularly attractive because of the diverse bioactive compounds they contain. *Lippia alba* has previously shown inhibitory activity against PoT, while garlic (*Allium sativum*) extracts and their bioactive compounds have demonstrated antifungal effects against several cereal pathogens, including *Magnaporthe*/*Pyricularia* spp. associated with blast diseases [9,10,11,12,13]. More recently, garlic extracts were shown to inhibit mycelial growth of *M. oryzae Triticum* pathotype under in vitro conditions, although their inhibitory effect decreased over time [14]. These findings support further evaluation of botanical products under seed and foliar application conditions in wheat.

Beneficial fungi also offer complementary mechanisms for suppressing fungal diseases. *Trichoderma* spp. are among the most extensively studied biocontrol fungi and can act through competition, antibiosis, mycoparasitism, and induction of plant defense, while also promoting plant growth [15,16]. Their potential against wheat blast has been demonstrated under in vitro and greenhouse conditions, including evidence of direct antagonism and mycoparasitism [17]. The saprophytic fungus *Epicoccum nigrum* has likewise shown biocontrol and plant-growth-promoting effects against blast disease in rice, but its potential in the wheat–PoT pathosystem remains poorly understood [18]. Thus, evaluating complementary fungal antagonists with different ecological and functional characteristics may expand the range of biological tools available for wheat blast management.

The interaction between biocontrol agents and the plant defense system is an additional mechanism of interest. Infection by *P. oryzae* can induce changes in reactive oxygen species metabolism and defense-related gene expression, while *Trichoderma*-mediated protection has been associated with the modulation of genes involved in pathogenesis-related responses, phenylpropanoid metabolism, and lipid signaling [19,20]. In the wheat–PoT pathosystem, defense-related gene expression also differs according to host susceptibility, with differences in the timing and persistence of PR-1 transcript accumulation associated with disease development [4]. These observations suggest that botanical and microbial treatments may suppress wheat blast not only through direct effects on the pathogen but also by enhancing or priming host defense responses. However, the combined effects of botanical extracts and antagonistic fungi on pathogen suppression, wheat growth, disease development, and defense-related gene expression have not been comprehensively evaluated in the wheat–PoT pathosystem.

Therefore, this study aimed to evaluate the antifungal and plant-beneficial effects of *Lippia alba* and *Allium sativum* extracts and the antagonistic fungi *Trichoderma harzianum* and *Epicoccum nigrum* against PoT using complementary in vitro and in vivo assays. Specifically, we assessed their effects on pathogen growth and conidiation, seed and seedling health, plant growth and vigor, disease development following seed and foliar applications, and, for the most effective treatment, the expression of selected defense-related genes in wheat seedlings. This integrated approach was used to identify candidate botanical and microbial treatments for further evaluation in wheat blast management and to provide insights into potential mechanisms underlying disease suppression.

## 2. Results

### 2.1. In Vitro Assays

#### 2.1.1. Effect of *Lippia alba* and *Allium sativum* Plant Extracts on PoT

All concentrations of *L. alba* extract significantly reduced the radial growth rate of all *Pyricularia oryzae Triticum* pathotype (PoT) isolates compared with the untreated control (UTC; *p* < 0.001; Supplementary Table S1). The strongest effects were observed at 1000 ppm, particularly for PY74 and PY07, while PY74 was also strongly inhibited at 500 ppm. Similarly, mycelial growth inhibition (MGI) increased with extract concentration, reaching 90.24% for PY74 and 72.31% for PY07 at 1000 ppm, and 64.86% for PY74 at 500 ppm (Figure 1A). Colony morphology and growth inhibition patterns are shown in Figure 1B.

Garlic (*Allium sativum*) extract also significantly reduced vegetative growth of all PoT isolates at all tested concentrations (Supplementary Table S2). The 3 and 5 mL treatments produced MGI values above 90% across all isolates, except PY15, which showed 56% inhibition at 3 mL (Figure 2A), without evident macroscopic changes in colony morphology (Figure 2B). The 3 and 5 mL treatments completely inhibited conidial production, indicating a strong effect on pathogen reproduction.

#### 2.1.2. Effect of *Trichoderma harzianum* and *Epicoccum nigrum* Antagonistic Fungi on PoT

Both *T. harzianum* isolates significantly reduced radial growth rate of the ten PoT isolates compared with the UTC (Supplementary Table S3). TH-FCCT-1155-4 showed the highest MGI, exceeding 50% against PY07 (57.00%), PY22 (56.97%), and PY82 (58.35%), whereas TH-FCCT-63 remained below 50%, with a maximum of 46.43% against PY02 (Figure 3A). Both *T. harzianum* isolates completely inhibited PoT sporulation, while also reducing mycelial growth to different extents among the pathogen–antagonist combinations (Supplementary Table S3). Direct hyphal interactions between *T. harzianum* and PoT were observed in the dual cultures. The differences in mycelial growth between the antagonist and PoT isolates may have contributed to the observed inhibition through rapid occupation of the available substrate (Figure 3B).

Both *E. nigrum* isolates affected PoT mycelial growth and conidial production, although the magnitude and direction of the response varied among pathogen isolates (Supplementary Table S4). EPICC-02 showed the strongest reduction in mycelial growth, with MGI values ranging from 30.0% to 68.9% and the highest inhibition against PY02 (68.9%; Figure 4A). EPICC-01 showed weaker activity, with MGI values below 50% across all PoT isolates, with the highest inhibition observed against PY82 (49.23%). The effect on conidial production was isolate-dependent. Conidial production increased significantly in several PoT isolates following treatment with EPICC-01 and/or EPICC-02, whereas it was significantly reduced in PY03 and showed no significant difference from the untreated control in some other combinations (Supplementary Table S4).

Regarding mycelial growth, both *E. nigrum* treatments resulted in significantly lower growth rates than the untreated control (UTC), although the magnitude of inhibition varied among PoT isolates (Supplementary Table S4). No visible inhibition zones were observed in the dual cultures, suggesting that competition for substrate may contribute to the observed antagonistic effect (Figure 4B).

### 2.2. In Vivo Assays

#### 2.2.1. Phytotoxicity Evaluation on Wheat Seeds

*L. alba* extract at 1000 ppm significantly reduced seed germination in both wheat cultivars and induced severe seedling stunting and distortion (Figure 5A,B). The 5% (*v*/*v*) propylene glycol vehicle control did not cause phytotoxic effects under the conditions evaluated. Based on these results, *L. alba* was subsequently evaluated at 500 ppm.

#### 2.2.2. Effect of Natural Substances on Wheat Growth and Seed-to-Seedling Transmission of PoT

In both wheat cultivars, seed treatments with *T. harzianum* TH-FCCT-1155-4, *E. nigrum* EPICC-02, and garlic extract were associated with higher values for several seedling growth parameters than the inoculated untreated control, particularly leaf number, stem thickness, total length, and dry biomass (Table 1 and Table 2). Garlic extract was associated with higher biomass and, in several combinations, greater longitudinal growth than the inoculated untreated control. In contrast, *L. alba* showed limited effects on seedling growth, although it significantly increased the greenness index in cv. ‘MS INTA 215’. The magnitude of these responses varied according to the PoT isolate and wheat cultivar.

Foliar disease severity was lower than in the inoculated untreated control in several treatment–isolate combinations in both cultivars, although the magnitude and statistical significance of the response varied among PoT isolates (Figure 6 and Figure 7). In cv. ‘Buck Aparcero’, disease severity was reduced most consistently by garlic extract and EPICC-02, while *L. alba* showed particularly strong efficacy against PY34 and PY82 despite its limited effect on plant growth. In cv. ‘MS INTA 215’, TH-FCCT-1155-4, EPICC-02, and garlic extract generally provided the strongest disease reduction, with TH-FCCT-1155-4 showing the greatest effect against PY82. In contrast, *L. alba* showed limited efficacy against PY22 in this cultivar.

#### 2.2.3. Control of PoT from Wheat Seeds Inoculated with Plant Extracts and Antagonists

Seed treatments affected subsequent foliar disease development differently depending on the PoT isolate (Figure 8). For the PY15 challenge, the overall effect of seed treatment was not statistically significant (ANOVA, F = 5.58, *p* = 0.0622), although garlic extract (5 mL) showed the lowest mean disease severity (6.2%), followed by *T. harzianum* TH-FCCT-1155-4 (11.8%), *Lippia* 500 ppm (16.0%), and EPICC-02 (18.8%), compared with 38.4% in the untreated control (UTC). For PY34, seed treatment had a significant effect on disease severity (ANOVA, F = 97.60, *p* = 0.0003). *T. harzianum* TH-FCCT-1155-4, *Lippia* 500 ppm, and garlic extract (5 mL) showed the lowest disease severity (10.5%, 13.0%, and 13.5%, respectively), and did not differ significantly from each other. EPICC-02 resulted in intermediate disease severity (25.5%), whereas the UTC showed the highest severity (51.5%) (Figure 8).

Lesion density showed a similar pattern (Figure 9). The treatments reduced disease severity in several isolate–treatment combinations, although the magnitude of the response varied among PoT isolates. For PY15, lesion density was significantly lower in all treated plants than in the UTC, with mean values ranging from 0.92 to 1.28 lesions cm^−2^ compared with 2.55 lesions cm^−2^ in the UTC (ANOVA, F = 18.66, *p* = 0.0075). For PY34, all treatments also significantly reduced lesion density relative to the UTC (3.57 lesions cm^−2^), with values ranging from 0.67 to 1.76 lesions cm^−2^ (ANOVA, F = 80.64, *p* = 0.0004). *T. harzianum* TH-FCCT-1155-4 showed the lowest lesion density for PY34 (0.67 lesions cm^−2^), consistent with its strong effect on disease severity. These results indicate that preventative seed treatments reduced the development of PoT lesions following subsequent foliar inoculation, although their effect on overall disease severity varied depending on the pathogen isolate.

#### 2.2.4. Control of PoT by Foliar and Spike Spray Application of Plant Extracts and Antagonists

##### Seedling-Stage Evaluation

The post-inoculation efficacy of the treatments varied according to cultivar and PoT isolate. In cv. ‘Buck Aparcero’, *T. harzianum* TH-FCCT-1155-4 showed the strongest response against PY02, markedly reducing AUDPC (18.2 vs. 175.7 in the UTC) and restricting symptoms to small, non-spreading lesions characteristic of a resistant-type interaction. EPICC-02, garlic extract (5 mL), and *L. alba* (500 ppm) also reduced disease, with *L. alba* showing particularly strong efficacy against PY15 (AUDPC: 38.75). Against PY22, all treatments significantly reduced AUDPC, whereas garlic extract showed the lowest values against PY34 and PY82. Across the five PoT isolates, *T. harzianum* and garlic extract reduced disease progression and final severity in several isolate–treatment combinations, although the magnitude of the response varied among isolates, with final severity values of 5.5–7.6% in some treatments compared with 30% in the UTC (Figure 10, Supplementary Table S5).

A similar pattern was observed in cv. ‘MS INTA 215’, although the magnitude of the treatment response varied among PoT isolates. *T. harzianum*, garlic extract, and *L. alba* were particularly effective against PY02, while all treatments reduced disease progression against PY15 and PY22. EPICC-02 showed the lowest AUDPC values against PY34 and PY82. Disease severity reductions varied among pathogen–cultivar combinations, with several treatments producing reductions exceeding 50% relative to the untreated control in specific combinations (Figure 11, Supplementary Table S6).

##### Spike-Stage Evaluation (Anthesis Post-Inoculation Efficacy and Thousand-Grain Weight)

Post-inoculation foliar applications at anthesis (GS 6.0) provided variable protection depending on cultivar and PoT isolate. In cv. ‘Buck Aparcero’, *T. harzianum* TH-FCCT-1155-4, garlic extract, and EPICC-02 reduced spike disease severity and were associated with higher thousand-grain weight (TGW) than the inoculated untreated control in several isolate–treatment combinations, although the magnitude of the response varied among PoT isolates. *T. harzianum* showed reduced spike severity and higher TGW than the UTC in several isolate–treatment combinations, whereas garlic extract showed strong effects against PY15 and PY82. Treatment effects on spike disease severity varied among PoT isolates. *T. harzianum*, garlic extract, and *E. nigrum* reduced disease severity in several isolate–treatment combinations, whereas the response to *L. alba* was more variable (Figure 12 and Figure 13).

Cv. ‘MS INTA 215’ showed greater susceptibility to wheat blast, and treatment efficacy varied more strongly among pathogen isolates. *T. harzianum*, garlic extract, and EPICC-02 reduced spike severity in several isolate–treatment combinations, although the magnitude of the response varied among PoT isolates. For several pathogen–cultivar combinations, these treatments were associated with higher TGW than the inoculated untreated control (Figure 14 and Figure 15). Overall, the results indicate that post-inoculation foliar applications at anthesis reduced spike blast severity in several pathogen–cultivar combinations and were associated with differences in TGW, although treatment responses varied according to the PoT isolate and wheat cultivar.

### 2.3. Evaluation of Defense Gene Expression (RT-qPCR)

Defense-related gene expression was evaluated using the PoT PY15–cv. ‘MS INTA 215’ pathosystem treated with garlic extract (5 mL). Garlic-treated seedlings showed higher transcript abundance of PR5 and APX than the corresponding untreated control at specific sampling times (Figure 16A,C). In contrast, WCI-2 and POX-2 did not show significant differences between treatments at the evaluated sampling times (Figure 16B,D). These results indicate that garlic treatment was associated with differences in the expression of selected defense- and oxidative stress-related genes during the wheat–PoT interaction.

## 3. Discussion

The present study provides evidence that botanical extracts and fungal antagonists can suppress PoT through effects on pathogen growth and reproduction, with additional treatment-associated responses observed at the plant level. The mechanisms underlying these effects appear to differ among treatments. The available evidence supports direct antifungal activity for the botanical extracts and direct antagonistic interactions for *T. harzianum*, whereas the contribution of plant-mediated responses was explored specifically for *A. sativum* through defense-related gene expression.

The antifungal activity of *Lippia alba* is consistent with previous reports of inhibitory effects of this botanical extract against PoT and other fungal pathogens [9,10]. The stronger inhibition observed at increasing concentrations suggests a concentration-dependent effect, although the present experiments do not allow the specific cellular or biochemical targets responsible for PoT growth inhibition to be identified. Differences from previously reported effective concentrations may reflect variation in extract composition, formulation, pathogen isolate, or experimental conditions. The phytotoxicity observed at 1000 ppm further indicates that the antifungal activity of *L. alba* must be considered together with its concentration-dependent effects on the host. At 500 ppm, *L. alba* showed variable effects on plant growth but reduced disease development following seed treatment. The variable efficacy of *L. alba* following application at anthesis may have been influenced by the formulation vehicle. Because the *L. alba* formulations contained 5% (*v*/*v*) propylene glycol and a vehicle-only control was not included in the spike disease assessment, the potential contribution of propylene glycol to the observed disease response cannot be determined. Therefore, the disease response observed following *L. alba* application should not be attributed exclusively to the botanical extract. Differences in tissue microenvironment and retention of volatile compounds may contribute to this contrasting response, although this hypothesis requires further investigation including a 5% (*v*/*v*) propylene glycol vehicle control under the same application conditions. To our knowledge, this study provides the first evidence of *L. alba* application against PoT under in vivo conditions in wheat.

Garlic extract showed strong antifungal activity among the plant-derived treatments. Its complete inhibition of PoT growth in vitro agrees with previous reports of the antifungal activity of *Allium sativum* against *P. oryzae* and other fungal pathogens [11,12,14]. This activity could be associated with sulfur-containing compounds, including allicin and related organosulfur metabolites, which have recognized antimicrobial properties [11]. The complete inhibition of conidiation observed in vitro further suggests that garlic treatment may interfere not only with vegetative growth but also with pathogen reproductive development. However, the present experiments did not identify the specific cellular targets or biochemical pathways affected by the extract. The absence of detectable phytotoxic effects is also consistent with previous observations in rice [13]. Importantly, the present study extends these findings by demonstrating garlic efficacy against PoT in wheat under in vivo conditions. Seed and foliar applications reduced disease development in several cultivar–isolate combinations, while garlic-treated plants also showed higher values for several growth parameters than the inoculated untreated control. However, because treated non-inoculated plants were not included, the present design does not allow us to distinguish direct effects of garlic extract on plant growth from growth responses associated with reduced disease development. Further experiments including treated non-inoculated plants would be required to determine whether garlic extract has a direct growth-promoting effect. Further physiological studies are also needed to determine whether the observed growth responses involve direct stimulation or modulation of plant metabolism.

The activity of *T. harzianum* appears to involve direct antagonistic interactions with PoT. In the dual-culture assays, direct hyphal interactions were observed, and the more rapid occupation of the available substrate by the antagonist may have contributed to pathogen growth suppression. Competition for nutrients and space and the production of antifungal metabolites are mechanisms commonly associated with *Trichoderma*-mediated antagonism [12,15,16]. However, because the specific metabolites and cellular interactions involved were not evaluated in the present study, these mechanisms should be considered as possible contributors rather than demonstrated modes of action.

Consistent with previous studies in wheat and other cereal systems [17,21,22], *T. harzianum* showed disease-suppressive effects in several experimental combinations and was associated with higher plant biomass and TGW in specific combinations. These responses may be consistent with mechanisms previously reported for *Trichoderma*-mediated disease suppression, including competition for nutrients and space, antibiosis, and plant-mediated effects [23,24]. However, the present study did not directly evaluate these mechanisms, and the absence of treated non-inoculated plants prevents us from distinguishing effects associated with disease suppression from direct effects on plant growth. The magnitude of the observed responses also varied among PoT isolates and wheat cultivars. Further experiments under standardized conditions are therefore required to determine the reproducibility and mechanisms underlying the observed effects.

*Epicoccum nigrum* also showed promising biocontrol activity, although its role in wheat blast remains less investigated. The reduction in PoT growth in vitro and disease suppression following seed treatment are consistent with previous findings in rice, where *E. nigrum* reduced blast development and promoted plant growth [18]. The absence of visible inhibition zones in the present study, together with the reduction in PoT mycelial growth, is compatible with a possible role of competition for space or nutrients in the observed antagonistic effect. However, other mechanisms, including the production of diffusible or volatile inhibitory compounds, were not directly evaluated. In addition, the effects of *E. nigrum* on PoT reproductive development were isolate-dependent. Although mycelial growth was reduced, conidial production increased significantly in several PoT isolates, whereas it was significantly reduced in PY03 and did not differ significantly from the untreated control in some other combinations. Increased conidial production may represent a potential epidemiological concern because conidia constitute an important source of secondary inoculum in blast diseases. However, as these observations were obtained under in vitro conditions, their implications for conidial dispersal, infection, and disease development under field conditions remain uncertain. The observed improvements in several growth-related parameters, reduced disease development, and higher TGW in specific experimental combinations further support the potential of *E. nigrum* for disease suppression, although these responses should be interpreted in relation to the isolate-dependent effects on pathogen reproduction. Because the underlying mechanisms were not directly evaluated, further studies are needed to determine the reproducibility, isolate dependence, and mechanisms contributing to *E. nigrum* activity in wheat.

The preventative seed-treatment experiment should be considered preliminary and exploratory because of the limited number of independent experimental units. Therefore, the observed treatment effects should be interpreted cautiously and confirmed in experiments with greater replication. A limitation of the anthesis experiments is that a non-inoculated control was not included; therefore, the observed differences in TGW should be interpreted relative to the inoculated untreated control and not as evidence of restoration to healthy-plant levels or preservation of total grain yield. A broader limitation of the present study is that all experiments were conducted under controlled in vitro or greenhouse conditions, using culture plates, rolled-towel assays, multi-pot trays, or pots. Although these experimental systems allowed controlled evaluation of treatment responses, they do not reproduce the environmental variability and epidemiological conditions encountered under field conditions. In addition, no registered fungicide was included as a reference treatment, and formulation stability, shelf life, residual activity, persistence or colonization of plants by the antagonistic fungi, and field efficacy were not evaluated. Therefore, the present results provide preliminary evidence for the selection of promising candidate treatments for further standardized greenhouse and field studies to evaluate their potential application in a wheat blast management programs.

The analysis of host defense responses provided additional evidence that garlic treatment may act beyond direct pathogen inhibition. In the susceptible ‘MS INTA 215’–PY15 interaction, garlic seed treatment was associated with increased transcript abundance of PR5 and APX at specific time points, whereas WCI-2 and POX-2 remained statistically unchanged. ROS production, antioxidant systems, and pathogenesis-related proteins are key components of plant responses to fungal infection [20]. The induction of APX is consistent with the involvement of ROS homeostasis and redox-regulatory mechanisms during wheat–PoT interactions. Likewise, the induction of PR5 is consistent with the activation of pathogenesis-related defense responses, as PR5 encodes thaumatin-like proteins that were shown to be transiently induced in wheat ears following infection with the *Magnaporthe* wheat blast pathogen [25,26]. The absence of significant changes in WCI-2 and POX-2 should not be interpreted as evidence that these genes are unrelated to the response, since defense signaling is highly dependent on tissue, genotype, and infection stage. Previous studies have likewise reported differential temporal regulation of defense-related genes in wheat–PoT interactions and following biological or chemical elicitation [4,19,27]. The present results therefore indicate an association between garlic treatment and changes in the expression of selected defense-related genes, although additional experiments using factorial designs, broader time-course analyses, and additional genes and genotypes are required to determine whether these changes reflect direct modulation of host defense responses.

## 4. Materials and Methods

### 4.1. Fungal Material

Ten isolates of *Pyricularia oryzae Triticum* pathotype (PoT), previously identified using molecular methods by Perelló et al., 2026 [28], were used in the in vitro assays with plant extracts and antagonistic fungi. Five isolates (PY02, PY15, PY22, PY34, and PY82) were subsequently selected for the in vivo experiments. All isolates are maintained in the fungal culture collection of the Centro de Investigaciones de Fitopatología (CIDEFI, FCAyF, UNLP).

### 4.2. In Vitro Assays

#### Evaluation of Plant Extracts and Antagonistic Fungi

*Lippia alba* (carvone chemotype) extract was provided by the Department of Biochemistry and Phytochemistry (National University of La Plata) as a formulated preparation containing 5% (*v*/*v*) propylene glycol. According to the information provided by the supplying laboratory, the stock extract concentration was 100,000 ppm (10%, *v*/*v*). The experimental formulations containing *L. alba* at 250, 500, and 1000 ppm were already formulated with propylene glycol and were applied directly in the corresponding treatments. No additional extraction, concentration, or reformulation procedure was performed by the authors. A 5% (*v*/*v*) propylene glycol solution was used as a vehicle control and processed under the same experimental conditions. The *L. alba* formulations were incorporated into 2% potato dextrose agar (PDA) using the poisoned food technique [29].

An aqueous garlic (*Allium sativum*) extract was supplied by Laboratorio Rapela (Argentina) as an experimental preparation obtained by hydrodistillation. The extract was used without further extraction or concentration. Volatile compounds were characterized by solid-phase microextraction coupled to gas chromatography–mass spectrometry (SPME-GC-MS) using an HP 6890 gas chromatograph coupled to a 5975C VL mass spectrometer (Agilent Technologies, Santa Clara, CA, USA). Diallyl disulfide was identified as the predominant volatile compound, accounting for 28.9% of the relative chromatographic peak area (Supplementary Table S7). The compounds were identified by comparison of their mass spectra with the NIST05 library and bibliographic data. Because no quantitative calibration with an authentic diallyl disulfide standard was performed, the GC-MS data were used for qualitative characterization only. The garlic extract was incorporated into 2% PDA at volumes of 1, 3, or 5 mL, and the final volume of each treatment was adjusted to 200 mL, corresponding to final extract concentrations of 0.5%, 1.5%, and 2.5% (*v*/*v*), respectively, using the poisoned food technique [29].

The antagonistic fungi included *Trichoderma harzianum* isolates TH-FCCT-1155-4 and TH-FCCT-63, molecularly identified and deposited in GenBank by Bader et al. (2020) [30], and supplied by INBIOTEC-CONICET, and *Epicoccum nigrum* isolates EPICC-01 and EPICC-02, morphologically identified and maintained at CIDEFI [31,32]. Antagonistic activity was evaluated by dual-culture assays on 2% PDA according to Bunbury-Blanchette and Walker (2019) [33]. The isolates were evaluated individually to account for isolate-dependent variation in antagonistic activity.

All in vitro assays were incubated for 7 days at 25 °C under a 12 h light/12 h dark photoperiod with white LED illumination. Four replicates were used for each extract concentration, antagonist, and pathogen isolate. Mycelial growth inhibition (MGI), sporulation capacity, and morphological alterations were evaluated. Untreated controls (UTC) consisted of pathogen cultures grown on PDA without treatment. A 5% (*v*/*v*) propylene glycol solution was included as a vehicle control for the *L. alba* treatments.

MGI was calculated as:

MGI (%) = [(Dc − Dt)/Dc] × 100

where Dc is the colony diameter of the untreated control and Dt that of the treatment.

### 4.3. In Vivo Assays

#### 4.3.1. Plant Material

Commercial wheat (*Triticum aestivum* L.) cultivars ‘MS INTA 215’ and ‘Buck Aparcero’, previously reported to differ in their responses to PoT, were used [4,28]. Wheat plants were maintained under controlled greenhouse conditions at 21 ± 2 °C with a 12 h photoperiod during the seedling experiments. For experiments conducted at anthesis, plants were maintained at 23 ± 2 °C under the same photoperiod.

#### 4.3.2. Phytotoxicity Evaluation

Based on the in vitro results, wheat seeds were treated with *L. alba* (1000 ppm), garlic extract (5 mL), *E. nigrum* EPICC-02 (2 × 10^5^ spores mL^−1^), or *T. harzianum* TH-FCCT-1155-4 (1 × 10^6^ spores mL^−1^) using the ISTA (2020) [34] rolled towel method. After 14 days, germination percentage and abnormal seedlings were recorded. Four replicates of 100 seeds were used per cultivar and treatment (400 seeds), with sterile distilled water as the UTC. A 5% (*v*/*v*) propylene glycol solution was included as a vehicle control for the *L. alba* treatment.

#### 4.3.3. Effect of Natural Treatments on Wheat Growth and Seed-to-Seedling Transmission of PoT

The efficacy of *L. alba*, garlic extract, *E. nigrum*, and *T. harzianum* in reducing PoT seed-to-seedling transmission and their association with growth-related responses in PoT-infected wheat seedlings were evaluated. Seeds were surface-disinfected with 0.5% NaClO, rinsed with sterile distilled water, and inoculated by immersion in a PoT conidial suspension (2 × 10^5^ spores mL^−1^) for 20 min at 150 rpm and 25 °C. After drying under a laminar flow hood, seeds were treated with the corresponding antagonist or extract.

Treatments were: PoT + *L. alba* (500 ppm), PoT + garlic (5 mL), PoT *+ E. nigrum* (2 × 10^5^ spores mL^−1^), and PoT + *T. harzianum* (1 × 10^6^ spores mL^−1^). PoT-inoculated untreated seeds (UTC+) and seeds treated with distilled water (UTC−) served as controls. A 5% (*v*/*v*) propylene glycol solution was included as a vehicle control for the *L. alba* treatment.

Seeds were sown in multi-pot trays containing autoclaved germination substrate and were monitored every 3 days until GS 1.5 [35]. Seedling dry weight, leaf number, total length, stem thickness, relative chlorophyll content (SPAD index), and disease severity were recorded. Chlorophyll measurements were obtained from the second and third leaves at GS 1.2–1.3 [35] using a Minolta SPAD 502 (Minolta, Osaka Japan) chlorophyll meter, while disease severity was quantified with ASSESS 2.0 software. Twenty seeds were individually treated for each cultivar/isolate combination and treatment, and subsequently were sown individually in 20 cells of multi-pot trays. Each cell containing one seedling was considered an experimental unit.

#### 4.3.4. Effect of Seed Treatments on Foliar Disease Caused by PoT

Cultivar ‘MS INTA 215’, selected for its high susceptibility to PoT, was used to evaluate the effect of seed treatments on subsequent foliar disease development. Unlike the seed-to-seedling transmission experiment described in Section 4.3.3, the seeds in this experiment were not inoculated with PoT prior to treatment. Seeds were treated with the corresponding antagonists or extracts as described in Section 4.3.3 and then sown in 20-L pots containing a 1:1 (*v*/*v*) mixture of soil and autoclaved sand. Plants were maintained in a greenhouse at 21 ± 2 °C under a 12 h photoperiod. At GS 1.5 [35], seedlings were foliar-inoculated with PoT isolates PY15 or PY34 (2 × 10^5^ spores mL^−1^) and maintained in a mist chamber for 48 h according to Martínez et al. (2019) [36].

At 14 dpi, disease severity and lesion density (lesions cm^−2^) were quantified using ASSESS 2.0. For each treatment and PoT isolate, two pots containing five plants each were used. The pot was considered the experimental unit, and the five plants within each pot were considered subsamples. Disease severity and lesion density values from the five plants within each pot were averaged before statistical analysis, resulting in two experimental units per treatment and PoT isolate. Untreated seeds subsequently inoculated with PoT at the foliar stage (UTC+) and untreated seeds sprayed with distilled water at the foliar stage (UTC−) served as controls. The two pots were considered independent replicates and were included as the replicate factor in the statistical analysis.

#### 4.3.5. Control of PoT by Foliar and Spike Application

Five PoT isolates (PY02, PY15, PY22, PY34, and PY82) were evaluated together with *T. harzianum* TH-FCCT-1155-4 and *E. nigrum* EPICC-02, selected based on their superior in vitro and phytotoxicity performance. Garlic extract was applied at 5 mL, whereas *L. alba* was used at 500 ppm because the 1000 ppm concentration was phytotoxic. The *L. alba* treatment consisted of the formulated 500 ppm extract containing 5% (*v*/*v*) propylene glycol and was applied directly without further reformulation. The treatments were applied by foliar spraying at the seedling (GS 1.5) and directly onto the spikes at anthesis (GS 6.0) stages [35]. For curative treatments, applications were performed 72 h after pathogen inoculation. Antagonists were applied as conidial suspensions at 2 × 10^5^ spores mL^−1^ (*E. nigrum*) and 1 × 10^6^ spores mL^−1^ (*T. harzianum*).

Experiments were conducted in 20-L pots under a 12 h photoperiod at 21 ± 2 °C (GS 1.5) or 23 ± 2 °C (GS 6.0). Disease severity was assessed as the percentage of symptomatic leaf area using ASSESS 2.0. Lesions were classified according to the scale proposed by Valent and Chumley [37], as adapted for wheat blast by Chavez and Kohli [5]. Disease severity and lesion density were recorded at 0, 7, and 14 dpi and were used to calculate the area under the disease progress curve (AUDPC). At 21 dpi, spike disease was assessed as the percentage of diseased spikelets. At maturity (GS 9.0) [35], thousand-grain weight was determined in the 2025 experiment. Ten plants were evaluated per cultivar/isolate combination and treatment, with each plant grown individually in a separate 20-L pot. Thus, each pot containing one plant was considered an independent experimental unit. The experiment was independently conducted in 2024 and 2025. For spike disease severity, Year and the Year × Treatment interaction was included in the initial statistical analysis. Because neither effect was significant, data from the two years were pooled for the final analysis.

### 4.4. Evaluation of Defense Gene Expression

Based on the in-planta results, the combination of PoT isolate PY15, wheat cultivar ‘MS INTA 215’, and garlic extract was selected for RT-qPCR analysis. Gene expression was evaluated following Portz et al. (2021) and Mogga et al. (2016) [27,38]. Leaves were collected at 0, 24, 48, and 72 h post-inoculation (hpi) and immediately frozen in liquid nitrogen. Three biological replicates were analyzed at each sampling time, with two technical replicates.

Total RNA was extracted using lysis and protein precipitation buffers followed by isopropanol precipitation, 70% ethanol washing, and resuspension in DEPC-treated water. RNA concentration was determined using a NanoDrop spectrophotometer and adjusted to 1 µg µL^−1^. Genomic DNA was removed with DNase I, and first-strand cDNA was synthesized using HindAnchorT primers and RevertAid Reverse Transcriptase according to the protocol described above.

The target genes were pathogenesis-related protein 5 (PR5), wheat chemically induced 2 (WCI-2), ascorbate peroxidase (APX), and peroxidase 2 (POX-2), using elongation factor 1-alpha (EF1α) as the reference gene. Primers and corresponding references are listed in Table 3.

For the RT-qPCR assays, a 48-well Applied Biosystems StepOne Real-Time PCR System (Thermo Fisher Scientific, Waltham, MA, USA) was used. Each individual reaction was performed in a final volume of 15 µL and contained 1× SYBR Green qPCR Master Mix (Thermo Fisher Scientific, Waltham, MA, USA), 0.5 µmol L^−1^ of each forward and reverse primer, and 0.2 µg of cDNA. Amplification consisted of an initial denaturation step at 95 °C for 10 min, followed by 40 cycles of 95 °C for 3 s and 60 °C for 30 s. A final melting-curve analysis was performed for each primer pair using the following conditions: 95 °C for 15 s, 60 °C for 1 min, and 95 °C for 15 s. The melting profiles were examined to assess the specificity of the amplification products. A no-template control (NTC) and a no-reverse-transcription (no-RT) control were included on all plates. Primer efficiency was determined using gene-specific standard curves. Relative transcript abundance was calculated using LinRegPCR software version 2021.1 and normalized against EF1α expression levels.

### 4.5. Statistical Analysis

Percentage data (MGI, germination, and disease severity) were arcsine square-root-transformed before analysis. Data from all in vitro and in vivo experiments were analyzed by ANOVA using InfoStat v. 2023 [42]. Normality and homogeneity of variance were assessed using Shapiro–Wilk or Kolmogorov–Smirnov and Levene’s tests, respectively, together with diagnostic plots. For the seed-treatment experiment described in Section 4.3.4, replicate (M1 and M2) was included in the statistical model together with treatment, using the mean values obtained from the five plants within each pot. For spike disease severity data obtained from experiments conducted independently in 2024 and 2025, Year, Treatment, and the Year × Treatment interaction were initially included in the ANOVA model. Because Year and Year × Treatment were not significant for either cultivar, data were pooled across years for the final analysis. When assumptions were not met, appropriate transformations were applied. If assumptions remained unmet, the Kruskal–Wallis test was used, as indicated in the corresponding figure legends. Tukey’s HSD test was used for multiple comparisons at *p *< 0.05.

For the RT-qPCR data, relative transcript abundance was analyzed separately for each target gene using a two-way ANOVA, with Treatment (untreated control and garlic extract) and Time (0, 24, 48, and 72 h post-inoculation) as fixed factors, including the Treatment × Time interaction. The three biological replicates were considered the experimental units, whereas technical replicates were averaged before statistical analysis. When significant effects or interactions were detected, means of the Treatment × Time combinations were compared using Tukey’s HSD test at *p* < 0.05.

## 5. Conclusions

Botanical extracts and beneficial fungi showed direct antifungal activity, disease-suppressive effects, and improved growth responses in PoT-infected wheat seedlings under the controlled conditions evaluated in this study. Among the evaluated treatments, *T. harzianum* and *E. nigrum* showed potential as biological control agents, while *A. sativum* showed antifungal and disease-suppressive effects across several in vitro and in vivo experimental combinations and was associated with increased transcript abundance of PR5 and APX at specific sampling times. These results identify *A. sativum*, *T. harzianum*, and *E. nigrum* as candidates for further standardized evaluation under greenhouse and field conditions for wheat blast management. Future studies should include appropriate non-inoculated treatment controls, reference fungicides, and assessments of formulation stability, shelf life, residual activity, persistence or plant colonization by antagonistic fungi, and efficacy under field conditions to further evaluate their potential integration into wheat blast management programs.

## Figures and Tables

**Figure 1:**
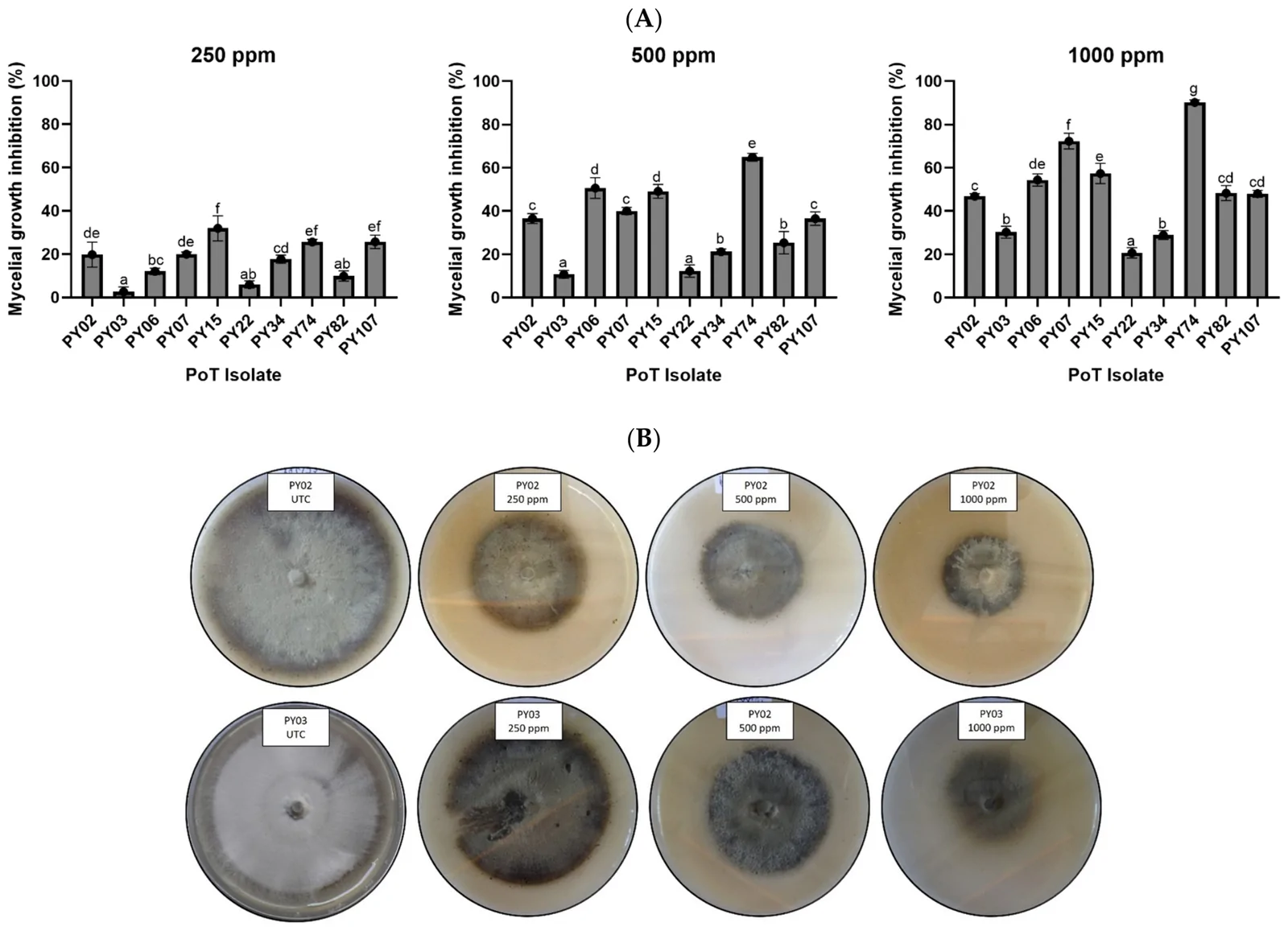
In vitro mycelial growth inhibition (MGI) and colony morphology of *Pyricularia oryzae Triticum* pathotype (PoT) isolates exposed to different concentrations of *Lippia alba* extract. (**A**) Percentage of MGI across ten PoT isolates treated with *L. alba* extract at 250, 500, and 1000 ppm after 7 days of incubation at 25 °C. Bars represent means ± SD (*n* = 4). Different lowercase letters indicate significant differences according to Tukey’s HSD test (*p *< 0.05). (**B**) Representative colony morphology of PoT isolates PY15 (**top row**) and PY34 (**bottom row**) on 2% potato dextrose agar (PDA) amended with 0 (UTC, untreated control), 250, 500, or 1000 ppm *L. alba* extract.

**Figure 2:**
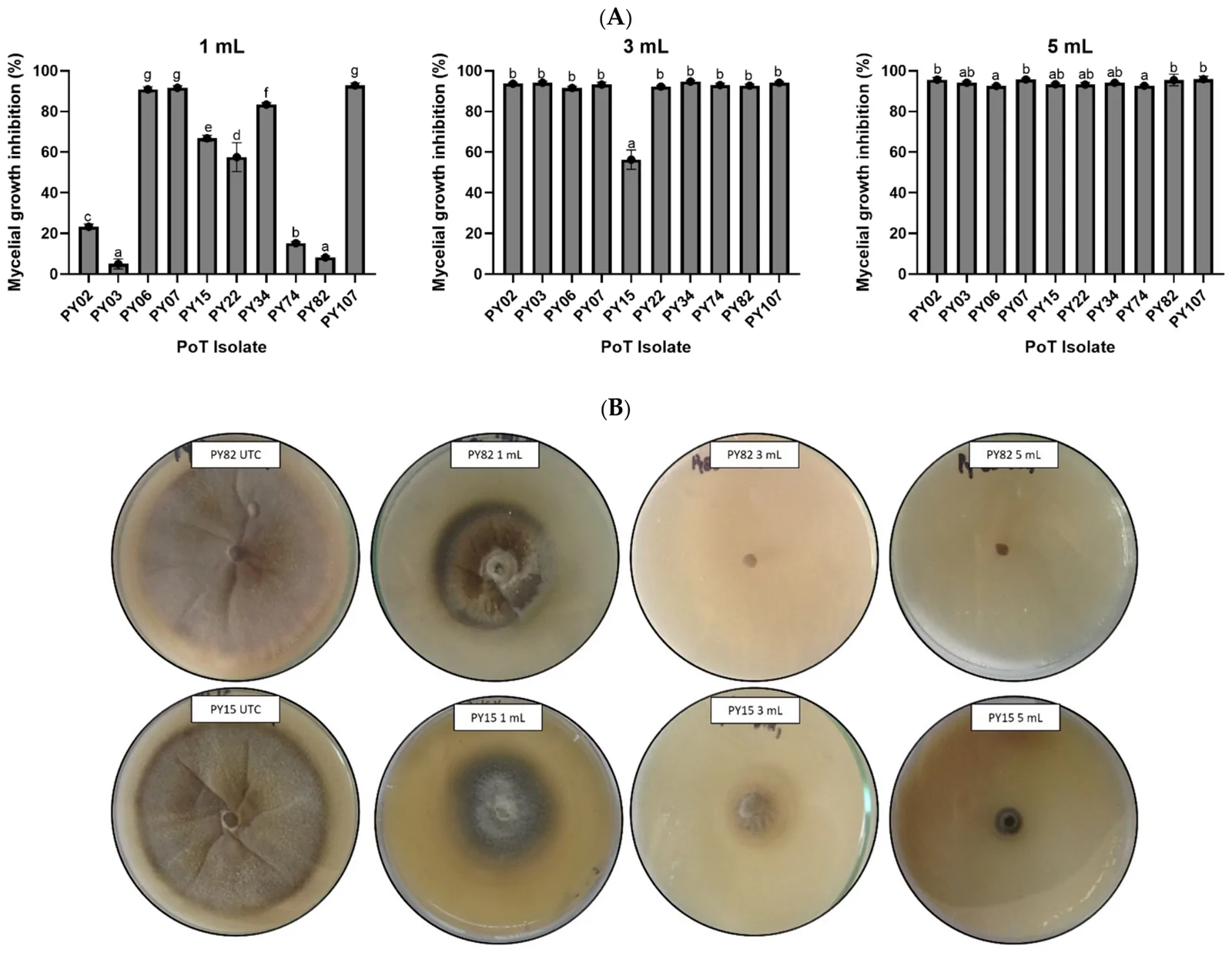
In vitro mycelial growth inhibition (MGI) and colony morphology of *Pyricularia oryzae Triticum* pathotype (PoT) isolates exposed to different concentrations of garlic (*Allium sativum*) extract. (**A**) Percentage of MGI across ten PoT isolates treated with 1, 3, and 5 mL of garlic extract after 7 days of incubation at 25 °C. Bars represent means ± SD (*n* = 4). Different lowercase letters indicate significant differences according to Tukey’s HSD test (*p *< 0.05). (**B**) Representative colony morphology of PoT isolates PY82 (**left panel**) and PY15 (**right panel**) on 2% potato dextrose agar (PDA) amended with 0 (UTC, untreated control), 1, 3, 5 mL of garlic extract.

**Figure 3:**
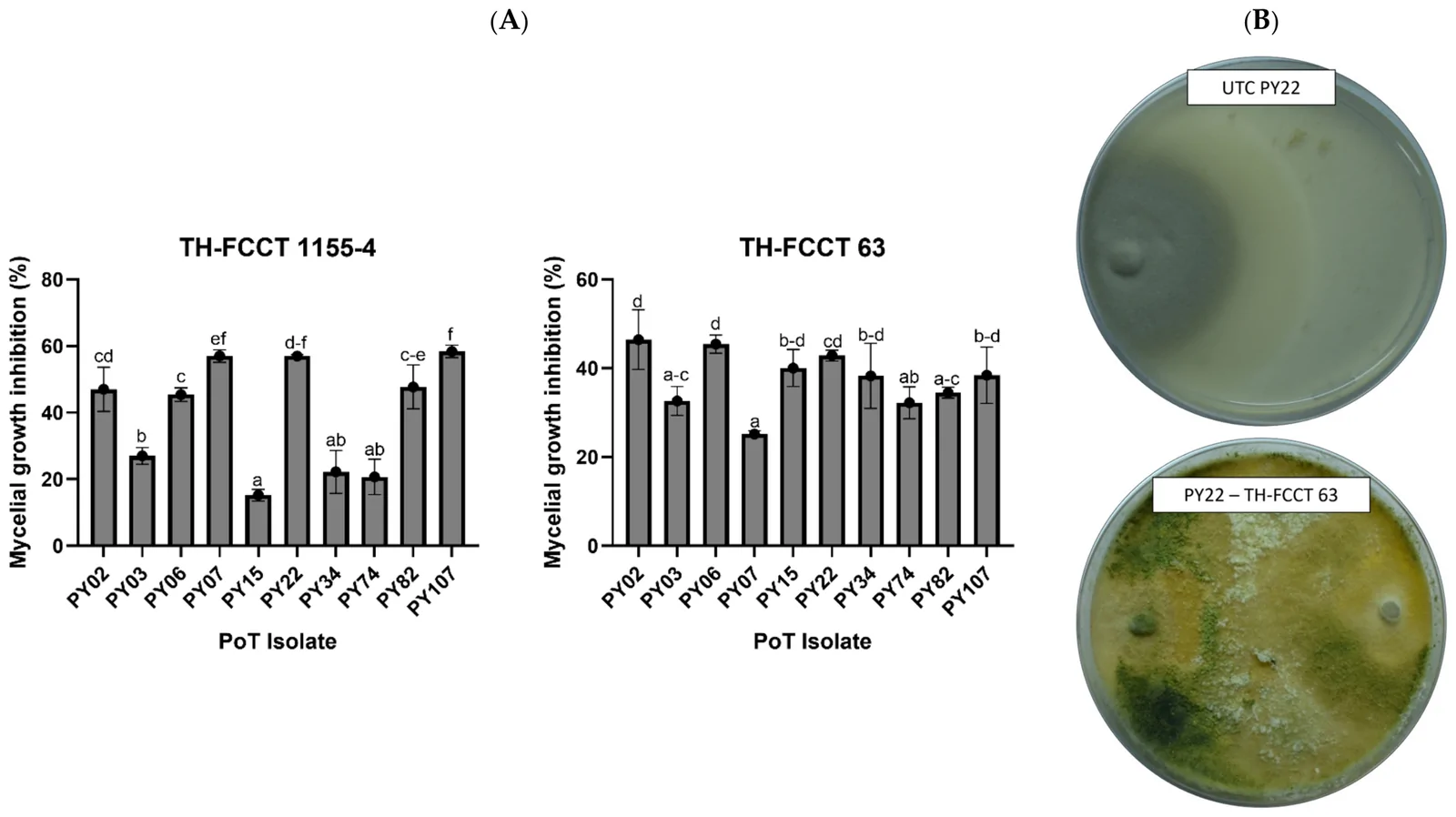
In vitro mycelial growth inhibition (MGI) and dual-culture interaction of *Trichoderma harzianum* against *Pyricularia oryzae Triticum* pathotype (PoT) isolates. (**A**) Percentage of MGI across ten PoT isolates in dual-culture assays with *T. harzianum* isolates TH-FCCT-1155-4 and TH-FCCT-63 after 7 days of incubation at 25 °C. Bars represent means ± SD (*n* = 4). Different lowercase letters indicate significant differences according to Tukey’s HSD test (*p *< 0.05). (**B**) Representative dual-culture plate showing the interaction between *T. harzianum* and PoT isolate PY15 (**top panel**) compared with the untreated control (**bottom panel**).

**Figure 4:**
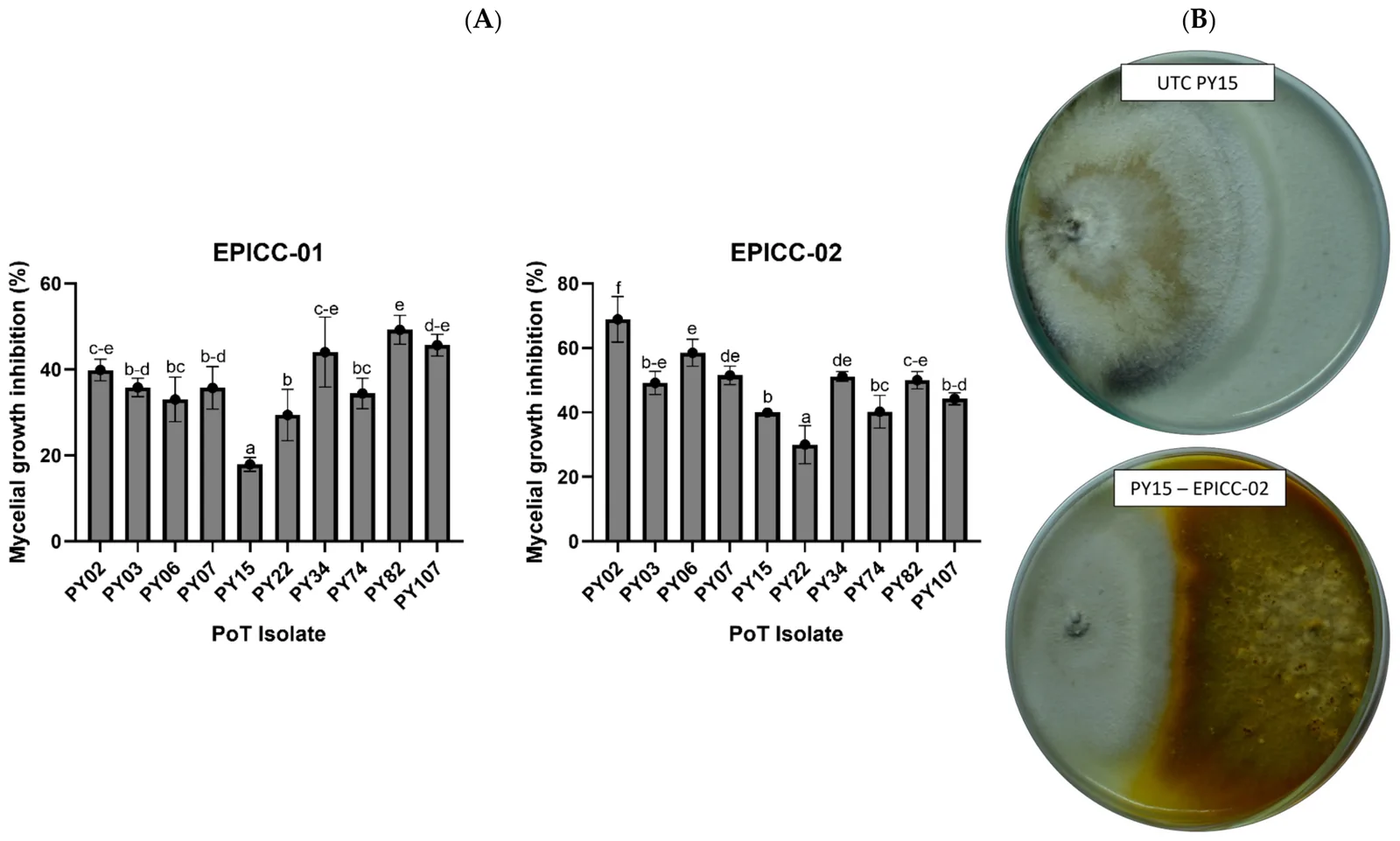
In vitro mycelial growth inhibition (MGI) and dual-culture interaction of *Epicoccum nigrum* against *Pyricularia oryzae Triticum* pathotype (PoT) isolates. (**A**) Percentage of MGI across ten PoT isolates in dual-culture assays with *E. nigrum* isolates EPICC-01 and EPICC-02 after 7 days of incubation at 25 °C. Bars represent means ± SD (*n* = 4). Different lowercase letters indicate significant differences according to Tukey’s HSD test (*p *< 0.05). (**B**) Representative dual-culture plates showing the interaction between *E. nigrum* and PoT isolates, characterized by the absence of visible inhibition zones.

**Figure 5:**
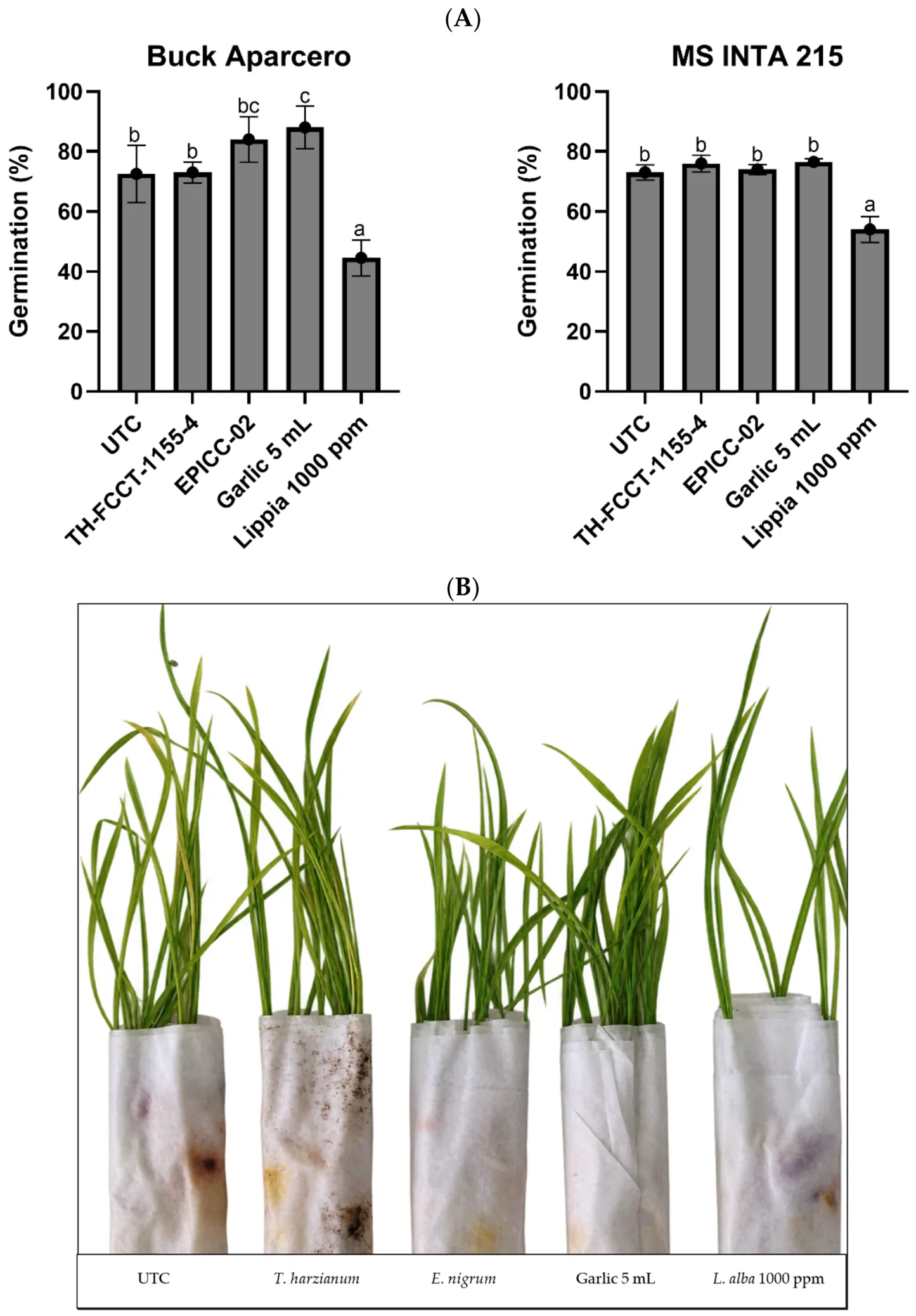
Phytotoxicity evaluation of biological and botanical seed treatments in wheat cultivars ‘Buck Aparcero’ and ‘MS INTA 215’ using the ISTA rolled towel paper method. (**A**) Germination percentage following seed treatment with sterile distilled water (UTC), *T. harzianum* TH-FCCT-1155-4, *E. nigrum* EPICC-02, garlic extract (5 mL), or *L. alba* extract (1000 ppm) after two weeks. Bars represent means ± SD (*n* = 4; 400 seeds per treatment–cultivar combination). Different lowercase letters indicate significant differences according to Tukey’s HSD test (*p *< 0.05). (**B**) Representative rolled-towel assay showing seedling development of cv. ‘Buck Aparcero’ following the different treatments.

**Figure 6:**
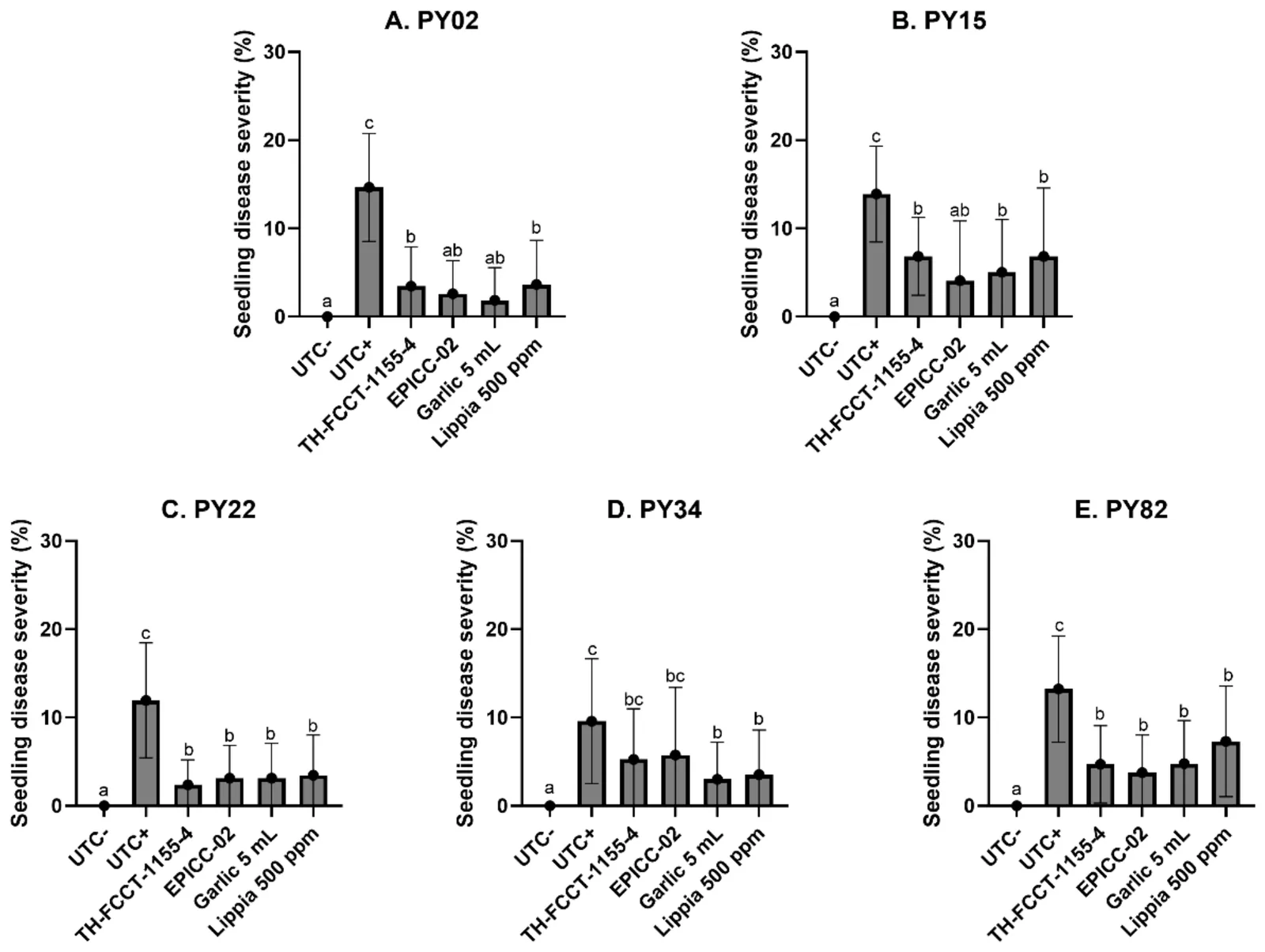
Effect of biological and botanical seed treatments on the seed-to-seedling transmission of *Pyricularia oryzae Triticum* pathotype (PoT) in wheat cv. ‘Buck Aparcero’. The panels illustrate disease severity across five representative pathogenic isolates: (**A**) PY02, (**B**) PY15, (**C**) PY22, (**D**) PY34, and (**E**) PY82. Seeds were inoculated with the respective pathogen isolate and treated with *T. harzianum* TH-FCCT-1155-4, *E. nigrum* EPICC-02, garlic extract (5 mL), or *L. alba* extract (500 ppm). Negative control (UTC−) represents non-inoculated healthy seedlings, whereas positive control (UTC+) indicates inoculated, non-treated seedlings. Disease severity was evaluated quantitatively on the fifth true leaf (*n* = 20 seedlings per treatment) as the percentage of affected leaf area using ASSESS 2.0 software. Vertical bars represent means ± SD values. Different lowercase letters indicate significant differences according to Tukey’s HSD test (*p *< 0.05).

**Figure 7:**
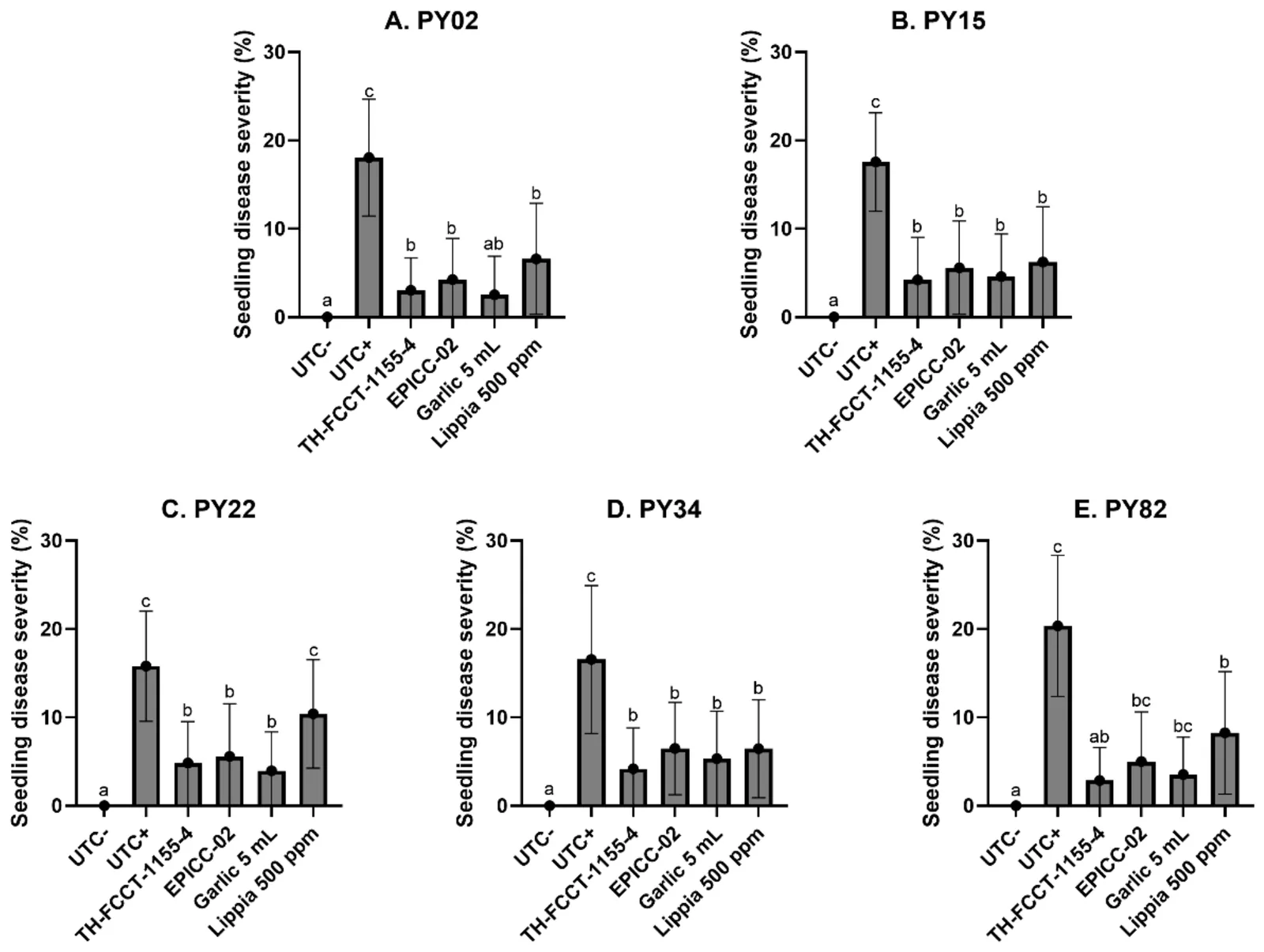
Effect of biological and botanical seed treatments on the seed-to-seedling transmission of *Pyricularia oryzae Triticum* pathotype (PoT) in wheat cv. MS INTA 215. The panels illustrate disease severity across five representative pathogenic isolates: (**A**) PY02, (**B**) PY15, (**C**) PY22, (**D**) PY34, and (**E**) PY82. Seeds were inoculated with the respective pathogen isolate and treated with *T. harzianum* TH-FCCT-1155-4, *E. nigrum* EPICC-02, garlic extract (5 mL), or *L. alba* extract (500 ppm). Negative control (UTC−) represents non-inoculated healthy seedlings, whereas positive control (UTC+) indicates inoculated, non-treated seedlings. Disease severity was evaluated quantitatively on the fifth true leaf (*n* = 20 seedlings per treatment) as the percentage of affected leaf area using ASSESS 2.0 software. Vertical bars represent means ± SD. Different lowercase letters indicate significant differences according to Tukey’s HSD test (*p *< 0.05).

**Figure 8:**
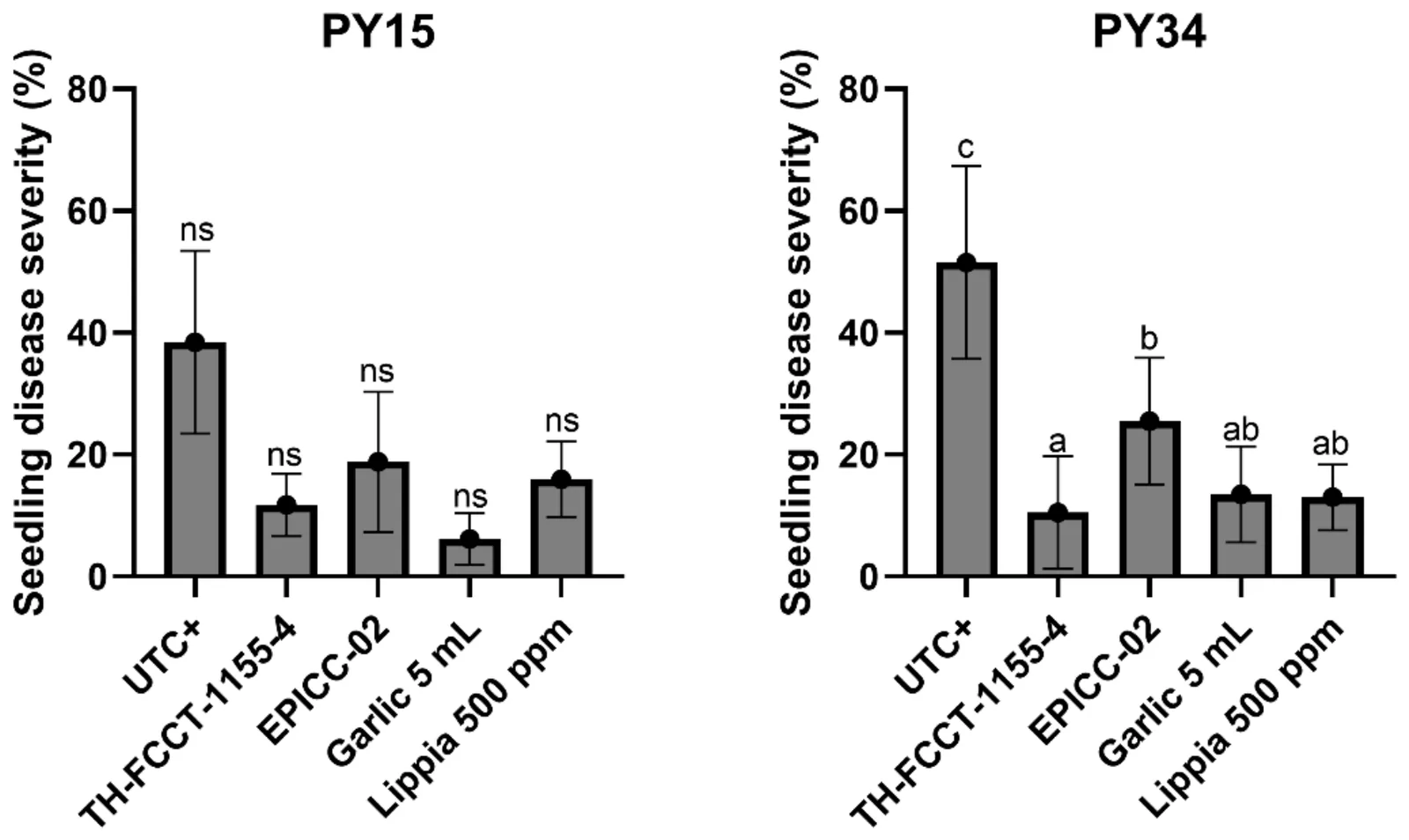
Effect of seed treatments with *T. harzianum* TH-FCCT-1155-4, *E. nigrum* EPICC-02, garlic extract, and *L. alba* extract on foliar disease severity caused by PoT isolates PY15 and PY34 in wheat cv. ‘MS INTA 215’. Seeds were treated with the respective antagonists or extracts and subsequently challenged with PoT at the seedling stage. Disease severity was evaluated at 14 dpi using ASSESS 2.0 and expressed as the percentage of symptomatic leaf area. Bars represent means ± SD of two experimental units (pots), each containing five plants. For statistical analysis, the mean severity of the five plants within each pot was used as the experimental observation. Different lowercase letters indicate significant differences among treatments within PY34 according to Tukey’s HSD test (*p *< 0.05). No significant overall treatment effect was detected for PY15 (ANOVA, *p* = 0.0622).

**Figure 9:**
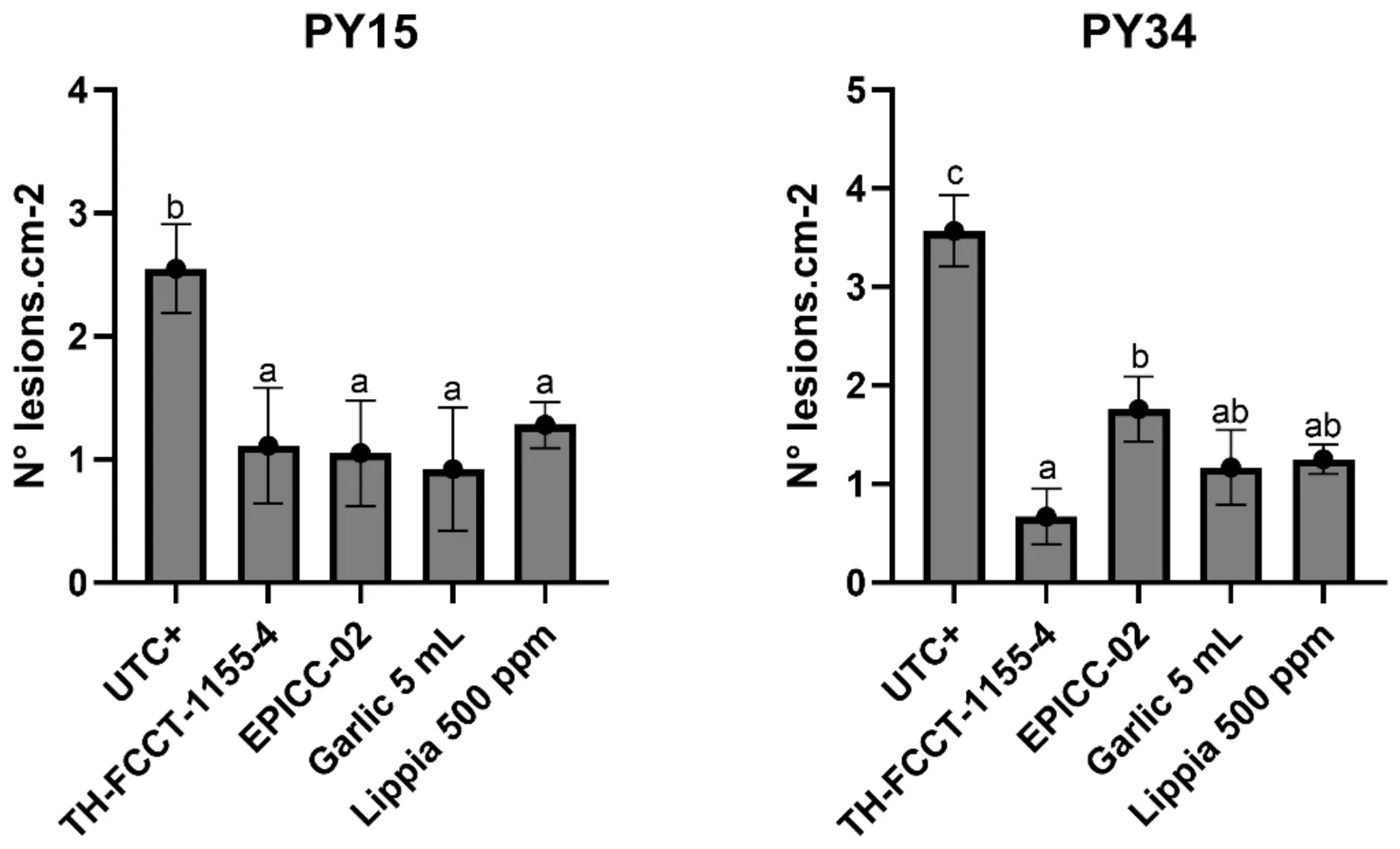
Effect of seed treatments with *T. harzianum* TH-FCCT-1155-4, *E. nigrum* EPICC-02, garlic extract, and *L. alba* extract on lesion density caused by PoT isolates PY15 and PY34 in wheat cv. ‘MS INTA 215’. Seeds were treated with the respective antagonist or extracts and subsequently challenged with PoT at the seedling stage. Lesion density was evaluated at 14 dpi using ASSESS 2.0 and expressed as the number of lesions cm^−2^. Bars represent means ± SD of two experimental units (pots), each containing five plants. For statistical analysis, the mean lesion density of the five plants within each pot was used as the experimental observation. Different lowercase letters indicate significant differences among treatments within each PoT isolate according to Tukey’s HSD test (*p *< 0.05).

**Figure 10:**
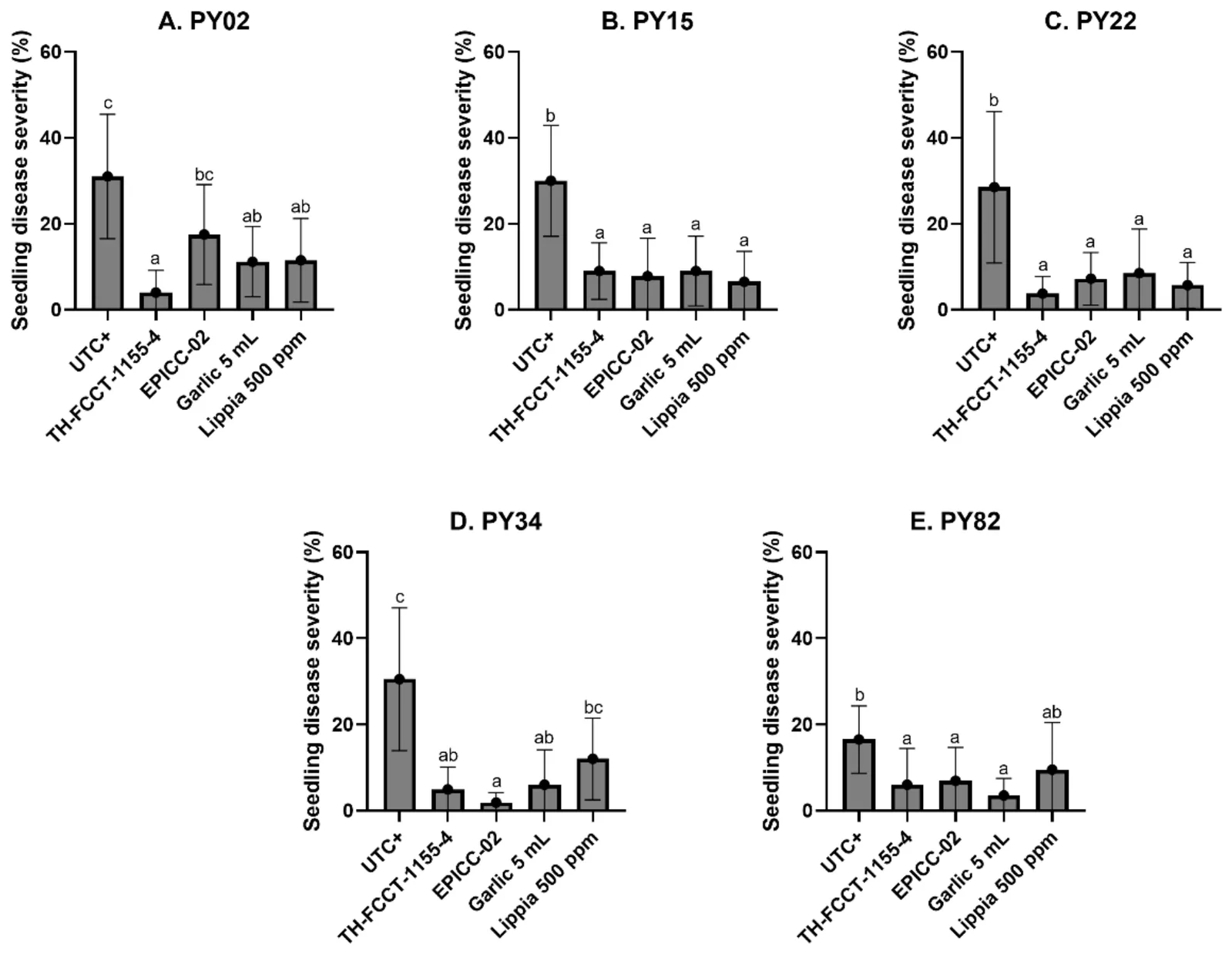
Post-inoculation efficacy of foliar applications of *T. harzianum* TH-FCCT-1155-4, *E. nigrum* EPICC-02, garlic extract (5 mL), and *L. alba* extract (500 ppm) against *Pyricularia oryzae Triticum* pathotype (PoT) isolates PY02 (**A**), PY15 (**B**), PY22 (**C**), PY34 (**D**), and PY82 (**E**) in wheat cv. ‘Buck Aparcero’. Disease severity was evaluated at the seedling stage and expressed as the percentage of symptomatic leaf area. Vertical bars represent means ± SD. Different lowercase letters indicate significant differences among treatments within each isolate according to Tukey’s HSD test (*p *< 0.05).

**Figure 11:**
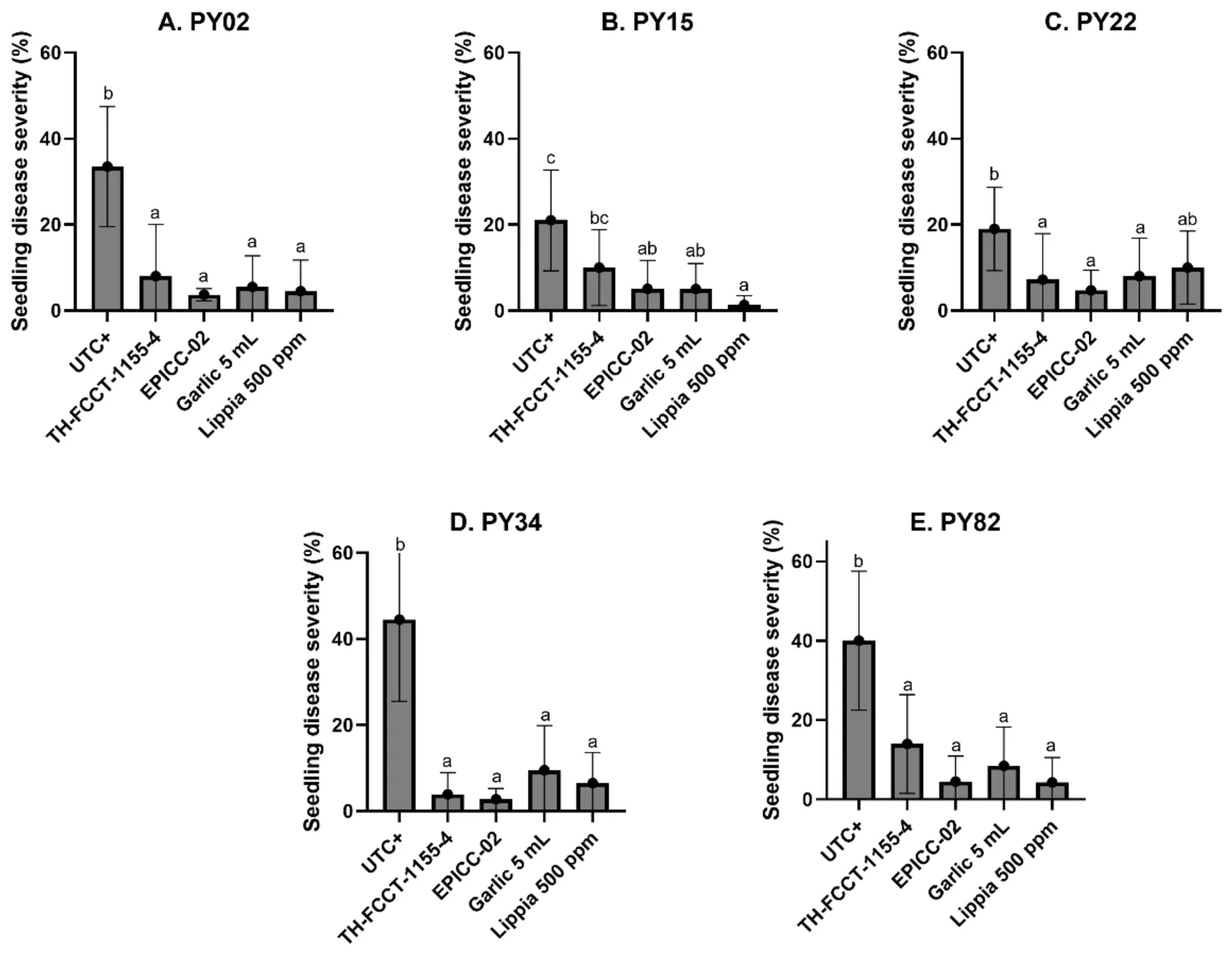
Post-inoculation efficacy of foliar applications of *T. harzianum* TH-FCCT-1155-4, *E. nigrum* EPICC-02, garlic extract (5 mL), and *L. alba* extract (500 ppm) against *Pyricularia oryzae Triticum* pathotype (PoT) isolates PY02 (**A**), PY15 (**B**), PY22 (**C**), PY34 (**D**), and PY82 (**E**) in wheat cv. ‘MS INTA 215’. Disease severity was evaluated at the seedling stage and expressed as the percentage of symptomatic leaf area. Vertical bars represent means ± SD. Different lowercase letters indicate significant differences among treatments within each isolate according to Tukey’s HSD test (*p *< 0.05).

**Figure 12:**
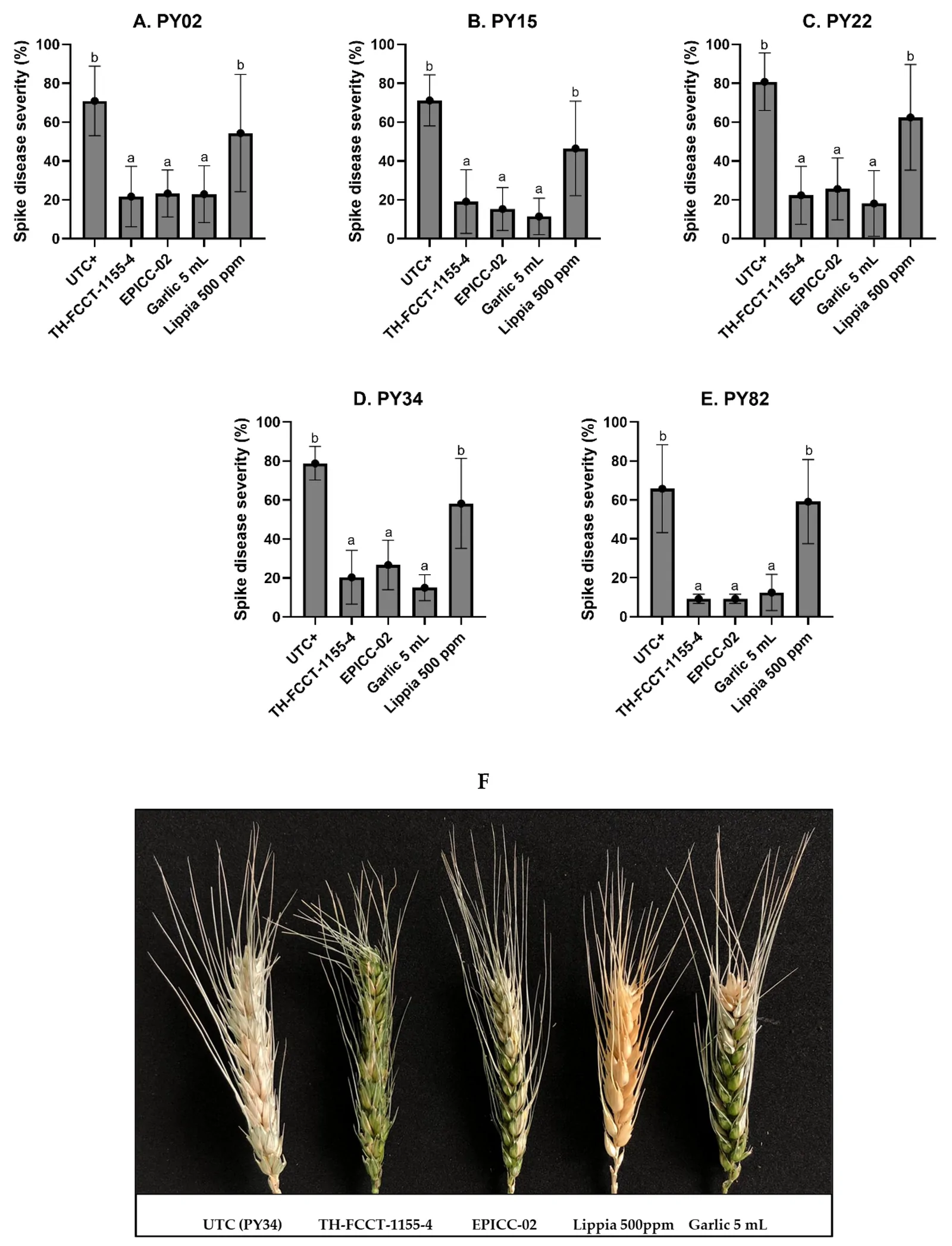
Effect of post-inoculation applications directly onto the spikes at anthesis (GS 6.0) of *T. harzianum* TH-FCCT-1155-4, *E. nigrum* EPICC-02, garlic extract (5 mL), and *L. alba* extract (500 ppm) on spike disease severity in wheat cv. ‘Buck Aparcero’ challenged with PoT isolates PY02 (**A**), PY15 (**B**), PY22 (**C**), PY34 (**D**), and PY82 (**E**). Spike severity was evaluated at 21 days post-inoculation and expressed as the percentage of diseased spikelets. Bars represent means ± SD (*n* = 20 spikes). Different lowercase letters indicate significant differences among treatments within each isolate according to Tukey’s HSD test (*p *< 0.05). Representative spike symptoms are shown below the graph (**F**).

**Figure 13:**
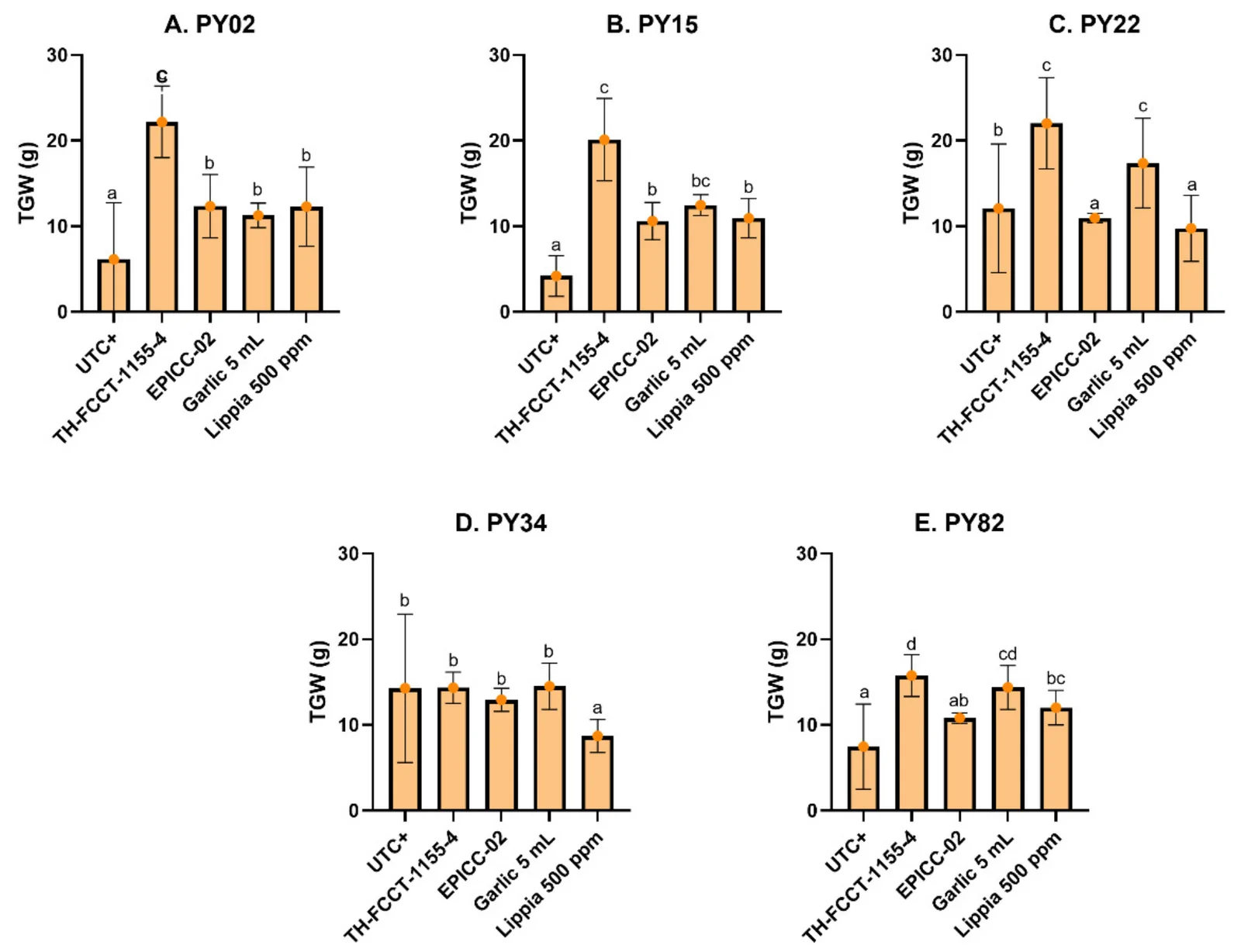
Effect of post-inoculation applications directly onto the spikes at anthesis of *T. harzianum* TH-FCCT-1155-4, *E. nigrum* EPICC-02, garlic extract (5 mL), and *L. alba* extract (500 ppm) on thousand-grain weight (TGW) of wheat cv. ‘Buck Aparcero’ challenged with PoT isolates PY02 (**A**), PY15 (**B**), PY22 (**C**), PY34 (**D**), and PY82 (**E**). Bars represent means ± SD (*n* = 10 spikes). Different letters indicate significant differences among treatments within each isolate (*p *< 0.05).

**Figure 14:**
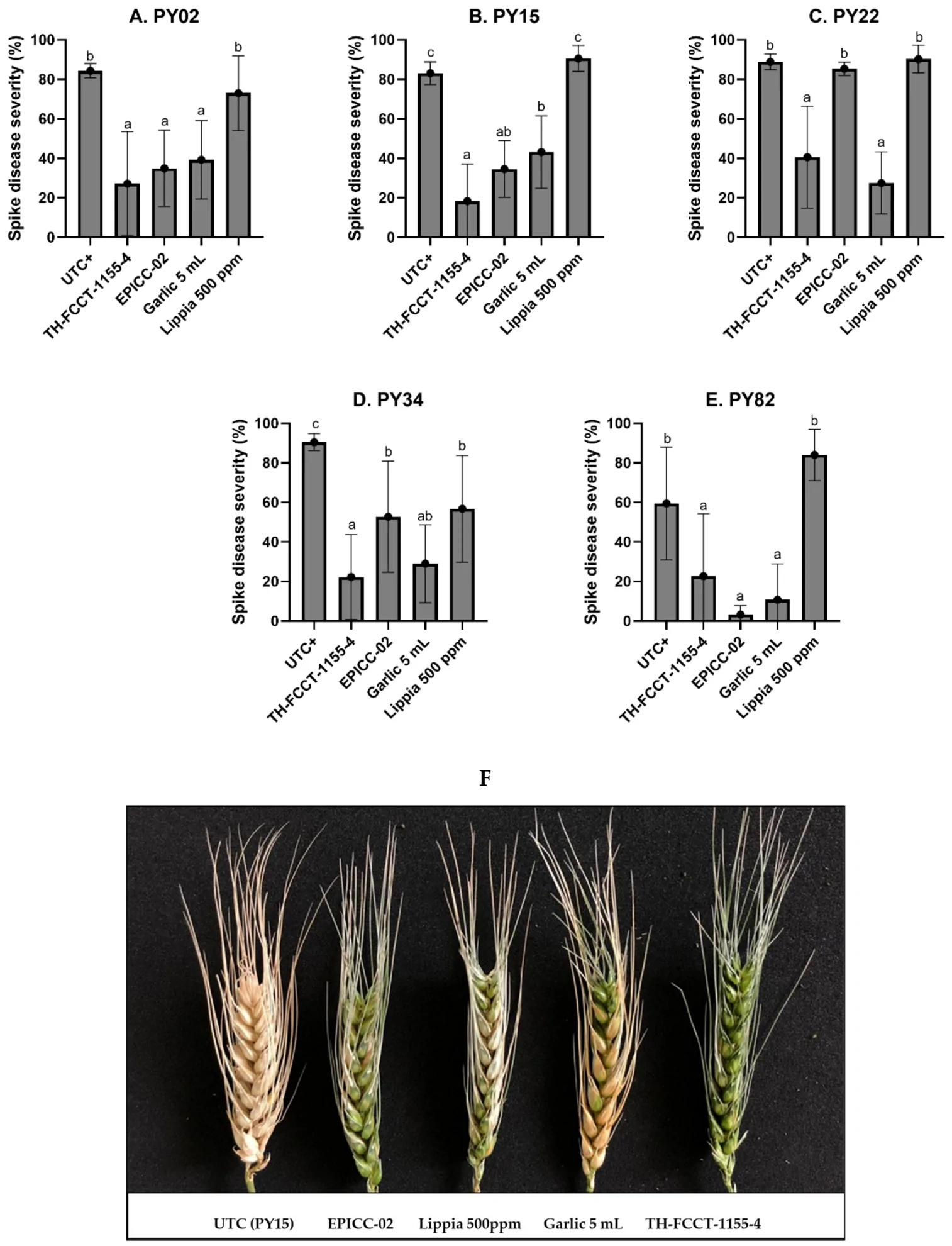
Effect of post-inoculation applications directly onto the spikes at anthesis (GS 6.0) of *T. harzianum* TH-FCCT-1155-4, *E. nigrum* EPICC-02, garlic extract (5 mL), and *L. alba* extract (500 ppm) on spike disease severity in wheat cv. ‘MS INTA 215’ challenged with PoT isolates PY02 (**A**), PY15 (**B**), PY22 (**C**), PY34 (**D**), and PY82 (**E**). Spike severity was evaluated at 21 days post-inoculation and expressed as the percentage of diseased spikelets. Bars represent means ± SD (*n* = 20 spikes). Different lowercase letters indicate significant differences among treatments within each isolate according to Tukey’s HSD test (*p *< 0.05). Representative spike symptoms are shown below the graph (**F**).

**Figure 15:**
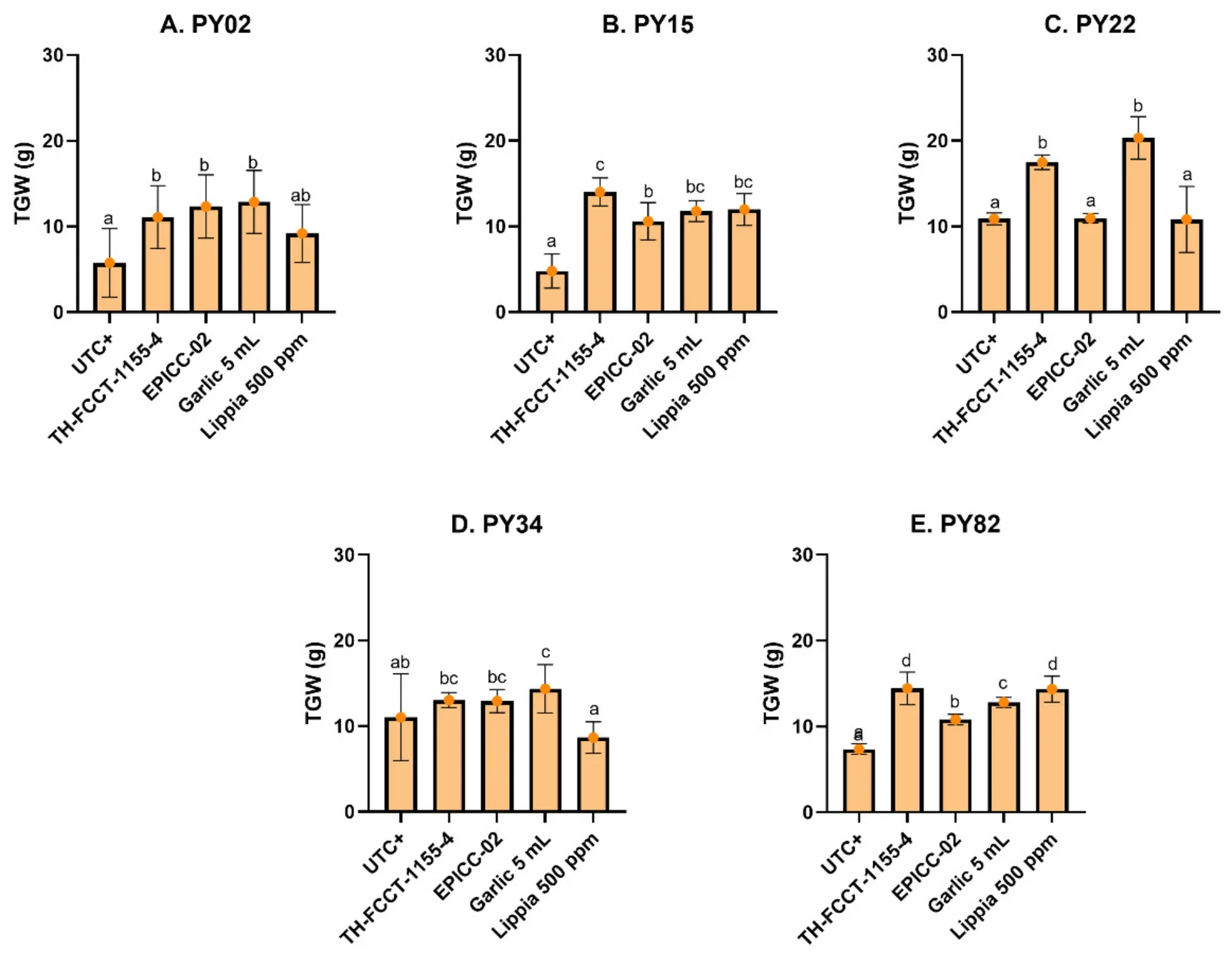
Effect of post-inoculation applications directly onto the spikes at anthesis of *T. harzianum* TH-FCCT-1155-4, *E. nigrum* EPICC-02, garlic extract (5 mL), and *L. alba* extract (500 ppm) on thousand-grain weight (TGW) of wheat cv. ‘MS INTA 215’ challenged with PoT isolates PY02 (**A**), PY15 (**B**), PY22 (**C**), PY34 (**D**), and PY82 (**E**). Bars represent means ± SD (*n* = 10 spikes). Different lowercase letters indicate significant differences among treatments within each isolate according to Tukey’s HSD test (*p *< 0.05).

**Figure 16:**
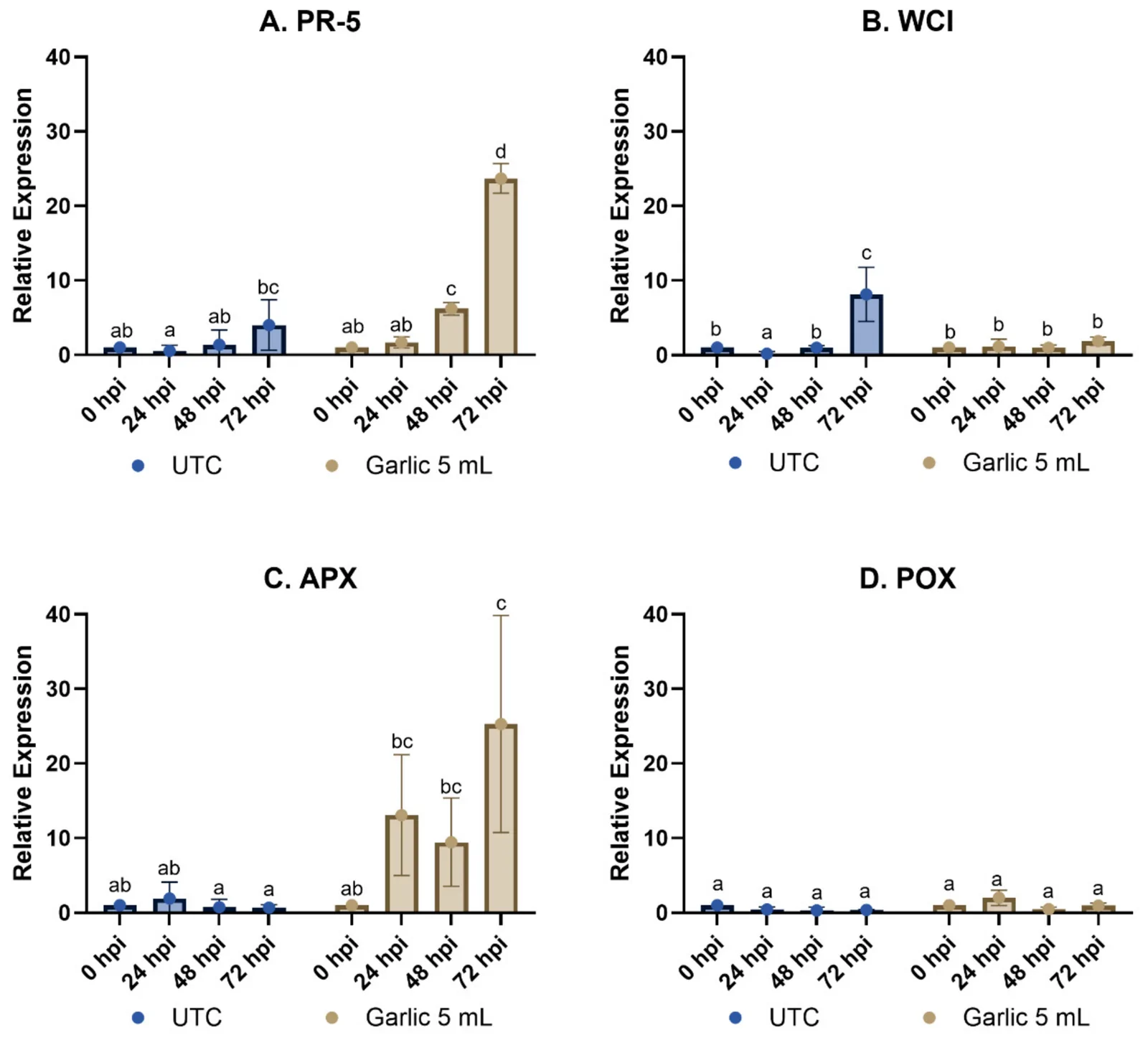
Relative expression of defense-related genes in the *P. oryzae Triticum* pathotype (PoT)–wheat cv. ‘MS INTA 215’ interaction following garlic extract seed treatment (5 mL). Relative transcript levels of (**A**) PR5, (**B**) WCI-2, (**C**) APX, and (**D**) POX-2 were determined by RT-qPCR at 0, 24, 48, and 72 h post-inoculation. The third leaf was inoculated with PoT at 2 × 10^5^ spores mL^−1^. Values represent means ± SD of three biological replicates. Technical replicates were averaged before statistical analysis. Different letters indicate significant differences among Treatment × Time combinations according to Tukey’s HSD test (*p* < 0.05).

**Table 1:** Effect of antagonists and plant extracts on the growth of Buck Aparcero wheat seedlings derived from seeds co-inoculated with five *Pyricularia oryzae Triticum* pathotype (PoT) isolates and antagonists or plant extracts. Different letters indicate statistically significant differences according to Tukey’s HSD test (*p* < 0.05).

ISOLATE	Treatment	Number of Leaves	Stem Thickness (mm)	Total Length (mm)	Greenness Index (SPAD Units)	Dry Weight (g)
PY02	UTC−	4.90 ab	1.81 a	54.91 a	30.09 a	0.18 a
	UTC+	4.55 a	1.89 ab	55.34 a	29.52 a	0.18 a
	TH-FCCT-1155-4	5.40 c	2.52 c	64.33 bc	31.07 a	0.22 ab
	EPICC-02	5.35 c	2.92 d	66.90 c	32.83 a	0.28 bc
	Garlic 5 mL	5.20 bc	2.74 cd	64.70 bc	36.72 b	0.23 bc
	Lippia 500 ppm	4.90 ab	2.17 b	60.15 ab	31.28 a	0.18 a
PY15	UTC−	4.90 b	1.81 a	54.91 a	30.09 b	0.18 ac
	UTC+	4.35 a	1.74 a	55.57 ab	25.74 a	0.14 a
	TH-FCCT-1155-4	5.30 b	2.55 bc	67.72 cd	31.34 b	0.23 cd
	EPICC-02	5.30 b	2.39 b	61.67 bc	32.03 b	0.21 bc
	Garlic 5 mL	5.15 b	2.92 c	68.00 d	31.35 b	0.27 d
	Lippia 500 ppm	4.25 a	1.90 a	56.10 ab	29.63 b	0.16 ab
PY22	UTC−	4.90 ab	1.81 a	54.91 a	30.09 a	0.18 a
	UTC+	4.75 a	1.94 a	53.19 a	32.23 ab	0.17 a
	TH-FCCT-1155-4	5.45 c	2.43 b	72.43 d	37.74 c	0.28 b
	EPICC-02	5.30 c	2.84 c	64.13 bc	35.06 bc	0.24 b
	Garlic 5 mL	5.20 bc	2.65 bc	65.78 cd	35.47 bc	0.25 b
	Lippia 500 ppm	4.90 ab	1.90 a	57.15 ab	31.49 a	0.17 a
PY34	UTC−	4.90 a	1.81 a	54.91 a	30.09 a	0.18 bc
	UTC+	4.75 a	1.72 a	60.82 bc	31.73 ab	0.13 a
	TH-FCCT-1155-4	5.30 b	2.80 bc	64.53 c	36.77 c	0.21 c
	EPICC-02	5.35 b	2.99 c	64.17 c	35.29 c	0.19 c
	Garlic 5 mL	5.30 b	2.54 b	63.38 bc	35.07 bc	0.21 c
	Lippia 500 ppm	5.10 ab	1.76 a	58.13 ab	31.28 a	0.14 ab
PY82	UTC−	4.90 a	1.81 a	54.91 b	30.09 ab	0.18 bc
	UTC+	4.85 a	1.45 a	46.63 a	28.76 a	0.07 a
	TH-FCCT-1155-4	5.80 c	2.37 b	68.49 c	32.02 bc	0.20 cd
	EPICC-02	5.75 bc	2.34 b	68.28 c	33.45 c	0.23 d
	Garlic 5 mL	5.40 b	2.30 b	55.29 b	33.09 c	0.22 cd
	Lippia 500 ppm	4.70 a	1.60 a	49.32 ab	29.28 a	0.14 b

**Table 2:** Effect of antagonists and plant extracts on the growth of MS INTA 215 wheat seedlings derived from seeds co-inoculated with five *Pyricularia oryzae Triticum* pathotype (PoT) isolates and antagonists or plant extracts. Different letters indicate statistically significant differences according to Tukey’s HSD test (*p *< 0.05).

ISOLATE	Treatment	Number of Leaves	Stem Thickness (mm)	Total Length (mm)	Greenness Index (SPAD Units)	Dry Weight (g)
PY02	UTC−	4.75 ab	1.72 a	51.76 a	28.20 a	0.14 ab
	UTC+	4.60 a	1.62 a	50.84 a	29.37 a	0.11 a
	TH-FCCT-1155-4	5.65 d	2.30 bc	61.78 b	30.43 a	0.18 c
	EPICC-02	5.55 cd	2.67 c	61.88 b	28.55 a	0.18 bc
	Garlic 5 mL	5.55 cd	2.61 c	63.88 b	30.21 a	0.25 d
	Lippia 500 ppm	5.20 bc	1.96 ab	52.64 a	27.63 a	0.12 a
PY15	UTC−	4.75 a	1.72 a	51.76 a	28.20 a	0.14 ab
	UTC+	4.85 a	1.82 a	53.8 ab	29.89 ab	0.14 ab
	TH-FCCT-1155-4	5.45 c	2.68 b	62.00 c	31.34 b	0.17 bc
	EPICC-02	5.55 c	3.17 c	58.70 bc	31.47 b	0.17 bc
	Garlic 5 mL	5.40 bc	2.46 b	56.25 bc	31.63 b	0.19 c
	Lippia 500 ppm	5.00 ab	1.58 a	51.50 a	32.03 b	0.12 a
PY22	UTC−	4.75 a	1.72 a	51.76 a	28.20 ab	0.14 bc
	UTC+	4.8 a	1.89 a	38.65 a	27.19 a	0.10 a
	TH-FCCT-1155-4	5.5 b	2.73 b	63.93 c	31.79 c	0.16 c
	EPICC-02	5.45 b	2.37 b	57.13 b	29.02 ac	0.20 d
	Garlic 5 mL	5.4 b	2.61 b	59.62 bc	31.48 bc	0.17 cd
	Lippia 500 ppm	5.3 b	1.77 a	32.12 a	30.24 ac	0.11 ab
PY34	UTC−	4.75 a	1.72 a	51.76 a	28.20 a	0.14 ab
	UTC+	4.75 a	2.09 a	55.37 a	30.99 b	0.13 a
	TH-FCCT-1155-4	5.65 b	2.76 b	65.40 b	31.90 b	0.16 bc
	EPICC-02	5.55 b	2.77 b	69.64 b	30.45 ab	0.19 cd
	Garlic 5 mL	5.65 b	2.56 b	67.40 b	31.48 b	0.20 d
	Lippia 500 ppm	5.05 a	2.56 b	57.05 a	32.16 b	0.12 a
PY82	UTC−	4.75 a	1.72 a	51.76 a	28.20 a	0.14 ab
	UTC+	4.90 ab	2.40 b	55.68 ab	30.68 ab	0.13 a
	TH-FCCT-1155-4	5.30 cd	2.82 bc	58.13 bc	39.19 c	0.23 cd
	EPICC-02	5.55 cd	2.68 b	66.70 d	40.06 c	0.21 d
	Garlic 5 mL	5.70 d	3.23 c	61.88 c	34.63 bc	0.17 b
	Lippia 500 ppm	5.20 bc	2.80 bc	57.26 b	32.78 ab	0.18 b

**Table 3:** Gene-specific primers used for the quantitative analysis of defense-related gene expression in wheat. The table lists the target genes, primer names, nucleotide sequences (5′–3′), and corresponding references. APX, ascorbate peroxidase; POX-2, peroxidase 2; WCI-2, wheat chemically induced 2; PR5, pathogenesis-related protein 5; EF1α, elongation factor 1-alpha.

Gene Target	Primer Name	Sequence (5′ → 3′)	Reference
*Ascorbate peroxidase*	APX_F APX_R	CTCCTACGGCGACCTCTACCAGC CTGAGGCTTGTCCTCCCTCCCTG	Gupta et al., 2021 [20].
*Peroxidase 2*	POX-2_F POX-2_R	CAACGACACCACCGACAACA GTCCATCACGAGTTCACCTT	Li et al., 2010 [39].
*Wheat chemically induced 2*	WCI-2_F WCI-2_R	ATCACGAGCCAGCTGCAAA GCCTTTTTCGCCTTGACATC	Sardesai et al., 2005 [40].
*Pathogenesis-related protein 5*	PR5_F PR5_R	TGCTCCTTCAATGGCGGTAG GTTGGGTGTTGGTAGGCTT	Ha et al., 2016 [25].
*Elongation factor 1-alpha*	EF1α_F EF1α_R	ATGATTCCCACCAAGCCCAT ACACCAACAGCCACAGTTTGC	McGrann et al., 2015 [41].

## Data Availability

Data are contained within the article and Supplementary Materials.

## Supplementary Materials

The following supporting information can be downloaded at: https://www.mdpi.com/article/10.3390/plants15213331/s1, Table S1: Mycelial growth rate of 10 PoT isolates treated with different concentrations of *Lippia alba* extract. Different lowercase letters indicate significant differences among treatments within each isolate according to Tukey’s HSD test (*p *< 0.05); Table S2: Mycelial growth rate of 10 PoT isolates treated with different concentrations of garlic extract. Different lowercase letters indicate significant differences among treatments within each isolate according to Tukey’s HSD test (*p *< 0.05); Table S3: Effect of two *T. harzianum* isolates on the mycelial growth rate of 10 PoT isolates. Different lowercase letters indicate significant differences among treatments within each PoT isolate according to Tukey’s HSD test (*p *< 0.05); Table S4: Effect of two *E. nigrum* isolates on the mycelial growth rate and sporulation of 10 PoT isolates. Different lowercase letters indicate significant differences among treatments within each PoT isolate according to Tukey’s HSD test (*p *< 0.05); Table S5: Effect of antagonist and plant extract treatments on AUDPC, lesion type, and lesions cm^−2^ in seedlings of the ‘Buck Aparcero’ cultivar challenged with five PoT isolates. Lesion types were classified according to the scale proposed by Valent and Chumley [38], as adapted for wheat blast by Chavez and Kohli [5]. Lesions cm^−2^ were quantified using Assess 2.0 software. Different lowercase letters indicate significant differences among treatments within each PoT isolate according to Tukey’s HSD test (*p *< 0.05); Table S6: Effect of antagonist and plant extract treatments on AUDPC, lesion type, and lesions cm^−2^ in seedlings of the ‘MS INTA 215’ cultivar challenged with five PoT isolates. Lesion types were classified according to the scale proposed by Valent and Chumley [38], as adapted for wheat blast by Chavez and Kohli [5]. Lesions cm^−2^ were quantified using Assess 2.0 software. Different lowercase letters indicate significant differences among treatments within each PoT isolate according to Tukey’s HSD test (*p *< 0.05); Table S7: Identification of volatile compounds in the aqueous garlic extract by gas chromatography–mass spectrometry (GC–MS). Volatile compounds were identified by comparison of their mass spectra with the NIST05 library and bibliographic data. Tr, retention time (min); A%, relative peak area (%); IK exp, experimental Kovats index; CAS, Chemical Abstracts Service registry number.
