# Supplementary material for: Botanical Extracts and Antagonistic Fungi for the Sustainable Management of Wheat Blast Caused by Pyricularia oryzae Triticum Pathotype
Source: Plants (Basel). 2026 Oct 28;15(21):3331. doi: 10.3390/plants15213331 (PMC13765828; doi:10.3390/plants15213331)
Supplement: Supplementary file 1 [file plants-15-03331-s001.zip › plants-4576966-supplementary.pdf]

Table S1: Mycelial growth rate of 10 PoT isolates treated with different concentrations of *Lippia alba* extract. Different lowercase letters indicate significant differences among treatments within each isolate according to Tukey's HSD test (p < 0.05).

Isolate	Treatment	Mycelial growth rate (mm day^{-1})	Isolate	Treatment	Mycelial growth rate (mm day^{-1})
PY02	1000 ppm	6.66 a	PY22	1000 ppm	8.04 a
	500 ppm	7.95 b		500 ppm	8.90 b
	250 ppm	10.05 c		250 ppm	9.54 c
	UTC	12.54 d		UTC	10.15 d
PY03	1000 ppm	7.41 a	PY34	1000 ppm	7.00 a
	500 ppm	9.48 b		500 ppm	7.75 b
	250 ppm	10.34 c		250 ppm	8.10 b
	UTC	10.62 c		UTC	9.84 c
PY06	1000 ppm	5.70 a	PY74	1000 ppm	0.82 a
	500 ppm	6.16 a		500 ppm	2.96 b
	250 ppm	10.98 b		250 ppm	6.26 c
	UTC	12.48 c		UTC	8.41 d
PY07	1000 ppm	2.61 a	PY82	1000 ppm	5.03 a
	500 ppm	5.64 b		500 ppm	7.26 b
	250 ppm	7.54 c		250 ppm	8.77 c
	UTC	9.41 d		UTC	9.73 d
PY15	1000 ppm	5.04 a	PY107	1000 ppm	5.66 a
	500 ppm	6.00 a		500 ppm	6.91 b
	250 ppm	8.04 b		250 ppm	8.09 c
	UTC	11.81 c		UTC	10.89 d

Table S2: Mycelial growth rate of 10 PoT isolates treated with different concentrations of garlic extract. Different lowercase letters indicate significant differences among treatments within each isolate according to Tukey's HSD test (p < 0.05).

Isolate	Treatment	Mycelial growth rate (mm day^{-1})	Isolate	Treatment	Mycelial growth rate (mm day^{-1})
PY02	5 mL	0.75 a	PY22	5 mL	0.75 a
	3 mL	0.79 a		3 mL	0.79 a
	1 mL	8.11 b		1 mL	3.54 b
	UTC	10.57 c		UTC	8.39 c
PY03	5 mL	0.75 a	PY34	5 mL	0.79 a

	3 mL	0.79 a		3 mL	0.89 a
	1 mL	10.54 b		1 mL	1.86 b
	UTC	11.07 c		UTC	12.50 c
PY06	5 mL	0.75 a	PY74	5 mL	0.75 a
	3 mL	0.79 a		3 mL	0.82 a
	1 mL	0.82 a		1 mL	6.57 b
	UTC	8.86 b		UTC	9.18 b
PY07	5 mL	0.75 a	PY82	5 mL	0.75 a
	3 mL	0.75 a		3 mL	0.75 a
	1 mL	0.79 a		1 mL	8.61 b
	UTC	9.43 b		UTC	9.32 c
PY15	5 mL	0.75 a	PY107	5 mL	0.75 a
	3 mL	3.75 b		3 mL	0.75 a
	1 mL	5.04 c		1 mL	0.79 a
	UTC	11.50 d		UTC	11.43 b

Table S3: Effect of two *T. harzianum* isolates on the mycelial growth rate of 10 PoT isolates. Different lowercase letters indicate significant differences among treatments within each PoT isolate according to Tukey's HSD test ($p < 0.05$).

Isolate	Treatment	Mycelial growth rate (mm day^{-1})	Isolate	Treatment	Mycelial growth rate (mm day^{-1})
PY02	FCCT 63	4.74 a	PY22	FCCT 1155-4	5.04 a
	FCCT 1155-4	4.65 a		FCCT 63	6.48 b
	UTC	8.84 b		UTC	11.61 c
PY03	FCCT 1155-4	5.79 a	PY34	FCCT 1155-4	7.64 a
	FCCT 63	5.50 b		FCCT 63	6.88 b
	UTC	8.20 c		UTC	10.19 c
PY06	FCCT 63	4.82 a	PY74	FCCT 63	3.38 a
	FCCT 1155-4	4.82 a		FCCT 1155-4	8.97 b
	UTC	8.92 b		UTC	11.50 c
PY07	FCCT 1155-4	3.95 a	PY82	FCCT 63	4.48 a
	FCCT 63	6.91 b		FCCT 1155-4	4.65 a
	UTC	9.25 c		UTC	7.52 b
PY15	FCCT 63	4.48 a	PY107	FCCT 63	3.77 a
	FCCT 1155-4	6.42 b		FCCT 1155-4	3.95 a

UTC	7.57 c	UTC	6.14 b

Table S4: Effect of two *E. nigrum* isolates on the mycelial growth rate and sporulation of 10 PoT isolates. Different lowercase letters indicate significant differences among treatments within each PoT isolate according to Tukey's HSD test ($p < 0.05$).

Isolate	Treatment	Mycelial growth rate (mm day^{-1})	Conidial concentration (conidia mL^{-1})	Isolate	Treatment	Mycelial growth rate (mm day^{-1})	Conidial concentration (conidia mL^{-1})
	EPICC-02	5.93 a	715000a		EPICC-02	6.00 a	832500b
PY02	EPICC-01	5.93 a	380000a	PY22	EPICC-01	6.00 a	825000b
	UTC	9.93 b	225000a		UTC	8.54 b	340000a
	EPICC-01	5.50 a	710000a		EPICC-02	4.40 a	750000b
PY03	EPICC-02	5.71 a	700000a	PY34	EPICC-01	5.04 a	787500c
	UTC	9.75 b	1625000b		UTC	9.08 b	250000a
	EPICC-02	3.32 a	252500a		EPICC-01	5.46 a	735000b
PY06	EPICC-01	5.36 b	252500a	PY74	EPICC-02	5.89 a	745000b
	UTC	8.04 c	216250a		UTC	8.57 b	237500a
	EPICC-02	4.97 a	800000b		EPICC-02	4.64 a	850000b
PY07	EPICC-01	5.36 a	762500b	PY82	EPICC-01	4.72 a	775000b
	UTC	8.75 b	275000a		UTC	9.39 b	50000a
	EPICC-02	5.71 a	757500b		EPICC-01	5.36 a	310000a
PY15	EPICC-01	7.04 b	750000b	PY107	EPICC-02	5.50 a	297500b
	UTC	9.11 c	212500^{a}		UTC	9.82 b	97500a

Table S5: Effect of antagonist and plant extract treatments on AUDPC. lesion type. and lesions cm^{-2} in seedlings of the 'Buck Aparcero' cultivar challenged with five PoT isolates. Lesion types were classified according to the scale proposed by Valent and Chumley [38]. as adapted for wheat blast by Chavez and Kohli [5]. Lesions.cm^{-2} were quantified using Assess 2.0 software. Different lowercase letters indicate significant differences among treatments within each PoT isolate according to Tukey's HSD test ($p < 0.05$).

Isolate	Treatment	AUDPC	Lesion type	Lesions.cm^{-2}
	UTC	175.70 c	4.70 b	4.00 c
	TH-FCCT-1155-4	18.20 a	0.70 a	0.80 a
PY02	EPICC-02	90.65 b	2.70 a	1.20 ab
	Garlic 5 mL	51.80 ab	1.50 a	2.20 ab
	Lippia 500 ppm	80.15 b	2.10 a	2.40 b

PY15	UTC	225.00 b	4.20 b	3.40 a
	TH-FCCT-1155-4	48.00 a	3.20 ab	1.80 a
	EPICC-02	47.70 a	2.10 a	2.40 a
	Garlic 5 mL	50.25 a	2.20 a	2.00 a
	Lippia 500 ppm	38.75 a	1.60 a	1.90 a
PY22	UTC+	183.75 b	5.00 b	3.10 a
	TH-FCCT-1155-4	22.70 a	1.50 a	1.40 a
	EPICC-02	36.30 a	1.60 a	2.60 a
	Garlic 5 mL	56.50 a	1.80 a	1.90 a
	Lippia 500 ppm	30.30 a	3.30 b	1.50 a
PY34	UTC+	189.50 c	4.00 c	3.60 b
	TH-FCCT-1155-4	45.85 ab	2.00 ab	1.10 a
	EPICC-02	27.10 ab	0.50 a	1.40 a
	Garlic 5 mL	24.00 a	1.10 ab	1.50 a
	Lippia 500 ppm	78.75 b	2.50 bc	2.80 ab
PY82	UTC+	102.00 b	3.00 a	2.80 b
	TH-FCCT-1155-4	42.75 a	1.00 a	1.00 a
	EPICC-02	44.10 a	1.90 a	2.40 ab
	Garlic 5 mL	21.50 a	2.20 a	1.10 a
	Lippia 500 ppm	65.00 ab	2.00 a	1.00 a

Table S6: Effect of antagonist and plant extract treatments on AUDPC. lesion type. and lesions cm^{-2} in seedlings of the 'MSINTA215 cultivar challenged with five PoT isolates. Lesion types were classified according to the scale proposed by Valent and Chumley [38]. as adapted for wheat blast by Chavez and Kohli [5]. Lesions.cm^{-2} were quantified using Assess 2.0 software. Different lowercase letters indicate significant differences among treatments within each PoT isolate according to Tukey's HSD test ($p < 0.05$).

Isolate	Treatment	AUDPC	Lesion type	Lesions.cm^{-2}
PY02	UTC	215.25 b	5.00 b	4.10 b
	TH-FCCT-1155-4	44.00 a	0.90 a	1.50 a
	EPICC-02	12.95 a	2.60 ab	1.70 a
	Garlic 5 mL	34.65 a	1.90 a	1.30 a
	Lippia 500 ppm	26.25 a	1.95 a	1.20 a
PY15	UTC	175.70 c	4.80 b	3.90 c
	TH-FCCT-1155-4	18.20 a	1.10 a	3.10 bc

	EPICC-02	90.65 b	1.20 a	1.60 ab
	Garlic 5 mL	51.80 ab	2.30 a	1.30 ab
	Lippia 500 ppm	80.15 b	0.70 a	0.80 a
PY22	UTC+	119.00 b	4.50 b	2.40 a
	TH-FCCT-1155-4	37.50 a	0.90 a	1.90 a
	EPICC-02	25.55 a	2.00 a	1.90 a
	Garlic 5 mL	42.70 a	1.30 a	1.40 a
	Lippia 500 ppm	49.00 a	1.30 a	1.50 a
PY34	UTC+	243.25 a	4.00 b	3.40 a
	TH-FCCT-1155-4	20.65 b	1.30 a	1.70 a
	EPICC-02	9.80 b	1.30 a	1.90 a
	Garlic 5 mL	64.75 b	1.30 a	3.00 a
	Lippia 500 ppm	48.85 b	1.30 a	2.80 a
PY82	UTC+	266.00 b	5.00 b	3.80 a
	TH-FCCT-1155-4	99.40 a	1.10 a	3.60 a
	EPICC-02	26.25 a	1.00 a	1.50 a
	Garlic 5 mL	54.25 a	0.50 a	2.90 a
	Lippia 500 ppm	29.08 a	1.10 a	1.80 a

Table S7: Identification of volatile compounds in the aqueous garlic extract by gas chromatography–mass spectrometry (GC–MS). Volatile compounds were identified by comparison of their mass spectra with the NIST05 library and bibliographic data. Tr. retention time (min); A%. relative peak area (%); IK exp. experimental Kovats index; CAS. Chemical Abstracts Service registry number.

Tr (min)	A%	IK exp	Identification	CAS#
2.19	1.2	687	Allyl mercaptan	870-23-5
6.84	0.5	857	Diallyl sulfide	592-88-1
8.48	1.1	915	Allyl methyl sulfide	2179-58-0
9.14	0.2	936	3-Thietane	10304-16-2
9.20	0.1	938	Methyl 1-propenyl disulfide	5905-47-5
13.49	28.9	1079	Diallyl disulfide	2179-57-9
13.95	0.8	1094	(Z)- Methyl 1-propenyl disulfide	122156-03-0
14.13	6.4	1100	(E)- Methyl 1-propenyl disulfide	122156-02-9
15.25	1.7	1138	Methyl allyl trisulfide	34135-85-8
16.70	8.7	1187	3-Vinyl-3.6-dihydro-1.2-dithiin	62488-52-2
17.45	21.1	1213	3-Vinyl-3.4-dihydro-1.2-dithiin	62488-53-3
19.90	23.0	1301	Diallyl trisulfide	2050-87-5
20.61	1.0	1329	di(1-propenyl) trisulfide	-
22.03	0.1	1383	Allyl methyl tetrasulfide	-
25.99	0.2	1541	Diallyl tetrasulfide	2444-49-7
